# Suppression of pancreatic ductal adenocarcinoma growth and metastasis by fibrillar collagens produced selectively by tumor cells

**DOI:** 10.1038/s41467-021-22490-9

**Published:** 2021-04-20

**Authors:** Chenxi Tian, Ying Huang, Karl R. Clauser, Steffen Rickelt, Allison N. Lau, Steven A. Carr, Matthew G. Vander Heiden, Richard O. Hynes

**Affiliations:** 1 Koch Institute for Integrative Cancer Research, Massachusetts Institute of Technology, Cambridge, MA USA; 2 Broad Institute of MIT and Harvard, Cambridge, MA USA; 3 Dana-Farber Cancer Institute, Boston, MA USA; 4 Howard Hughes Medical Institute, Chevy Chase, MD USA

**Keywords:** Cancer microenvironment, Pancreatic cancer, Metastasis

## Abstract

Pancreatic ductal adenocarcinoma (PDAC) has a collagen-rich dense extracellular matrix (ECM) that promotes malignancy of cancer cells and presents a barrier for drug delivery. Data analysis of our published mass spectrometry (MS)-based studies on enriched ECM from samples of progressive PDAC stages reveal that the C-terminal prodomains of fibrillar collagens are partially uncleaved in PDAC ECM, suggesting reduced procollagen C-proteinase activity. We further show that the enzyme responsible for procollagen C-proteinase activity, bone morphogenetic protein1 (BMP1), selectively suppresses tumor growth and metastasis in cells expressing high levels of COL1A1. Although BMP1, as a secreted proteinase, promotes fibrillar collagen deposition from both cancer cells and stromal cells, only cancer-cell-derived procollagen cleavage and deposition suppresses tumor malignancy. These studies reveal a role for cancer-cell-derived fibrillar collagen in selectively restraining tumor growth and suggest stratification of patients based on their tumor epithelial collagen I expression when considering treatments related to perturbation of fibrillar collagens.

## Abstract (web summary)

Pancreatic ductal adenocarcinoma has a collagen-rich dense extracellular matrix that promotes malignancy of cancer cells. Here, the authors show that fibrillar collagen that is cancer-cell-derived, but not stroma-derived, selectively restrains tumor growth under control of their pC-proteinase, BMP1.

## Introduction

Pancreatic ductal adenocarcinoma (PDAC) is among the most lethal cancers with an overall 5-year survival rate of 9%^1^. PDAC is characterized by a pronounced resistance to radiation, cytotoxic, and molecular-targeting therapies^2^. This resistance of PDAC to therapy is suggested to be partly mediated by its prominent stroma, predominantly composed of extracellular matrix (ECM)^3^. PDAC ECM plays pleiotropic roles in promoting tumor malignancy and resistance to drugs; it acts directly on cancer cells through mechano-sensing receptors (integrins) to promote proliferation and locomotion; biochemically it provides a reservoir for cytokines and growth factors to promote cancer cell survival, proliferation, and metastasis, as well as fostering chemo-resistance by protecting cancer cells from apoptosis. Furthermore, the abundant ECM and water retention caused by proteoglycans and hyaluronan can lead to high interstitial fluid pressure, which results in compression of the vasculature and impedes drug delivery^4–6^. However, non-selective depletion of stroma in mouse PDAC models, either by inhibiting ECM-inducing sonic hedgehog (SHH) signaling^7^ or by depleting α-smooth-muscle-actin-positive fibroblasts^8^, resulted in more aggressive cancer cells and poor survival, despite enhanced drug uptake. Similarly, clinical trials targeting metastatic PDAC by blocking SHH signaling resulted in paradoxical acceleration of disease progression leading to early termination of trials^9^.

The composition of tumor ECM has been systematically profiled by mass spectrometry (MS)-based proteomics on enriched ECM in both mouse models and human patients^10,11^. The matrisome comprises both core ECM proteins, such as collagens, glycoproteins, and proteoglycans, and ECM-associated proteins, such as ECM regulators, ECM-affiliated proteins, and secreted factors^12^. As expected for a desmoplastic stroma, matrisome proteins are upregulated as PDAC progresses^10,11^.

Fibrillar collagens (e.g. collagen I and III) are the most abundant ECM proteins in the PDAC ECM, comprising over 80% of all ECM mass^11^. Fibrillar collagens are generally suggested to be pro-tumorigenic; for instance, they provide a scaffold and reservoir for soluble growth factors^13^; their alignment, crosslinking, and remodeling could signal to cancer and stromal cells to promote pro-tumorigenic behaviors, such as proliferation, angiogenesis, invasion, metastasis, resistance to apoptosis and escape from dormancy^14^; and they also serve as a nutrient source for cancer cells to scavenge^15^. Collagen fibrils are largely synthesized and secreted by cancer-associated fibroblasts (CAFs)^16^, although cancer cells also deposit a small fraction of the total tumor collagen^11^. However, the significance and function of the cancer-cell-derived collagens has been largely overlooked.

In this study, we were interested to uncover ECM changes during PDAC progression beyond protein-expression levels. By further analyzing our published quantitative MS results on samples from progressive stages of human PDAC^11^, we discovered an increase in retention of the C-prodomains of fibrillar collagens, which suggested reduced pC-proteinase activity, in PDAC tumor ECM. We then showed that BMP1 (bone morphogenetic protein 1), the responsible pC-proteinase, promotes the production and assembly of fibrillar collagens. Furthermore, the effect of BMP1 on tumor progression and metastasis depends upon the level of cancer-cell-derived collagens. Our study thus identifies an unexpected role of ECM including cancer-cell-derived fibrillar collagens that is independent of stromally derived fibrillar collagen.

## Results

### Reduced removal of C-prodomain from fibrillar procollagens is a characteristic of PDAC ECM

**Fig. 1:**
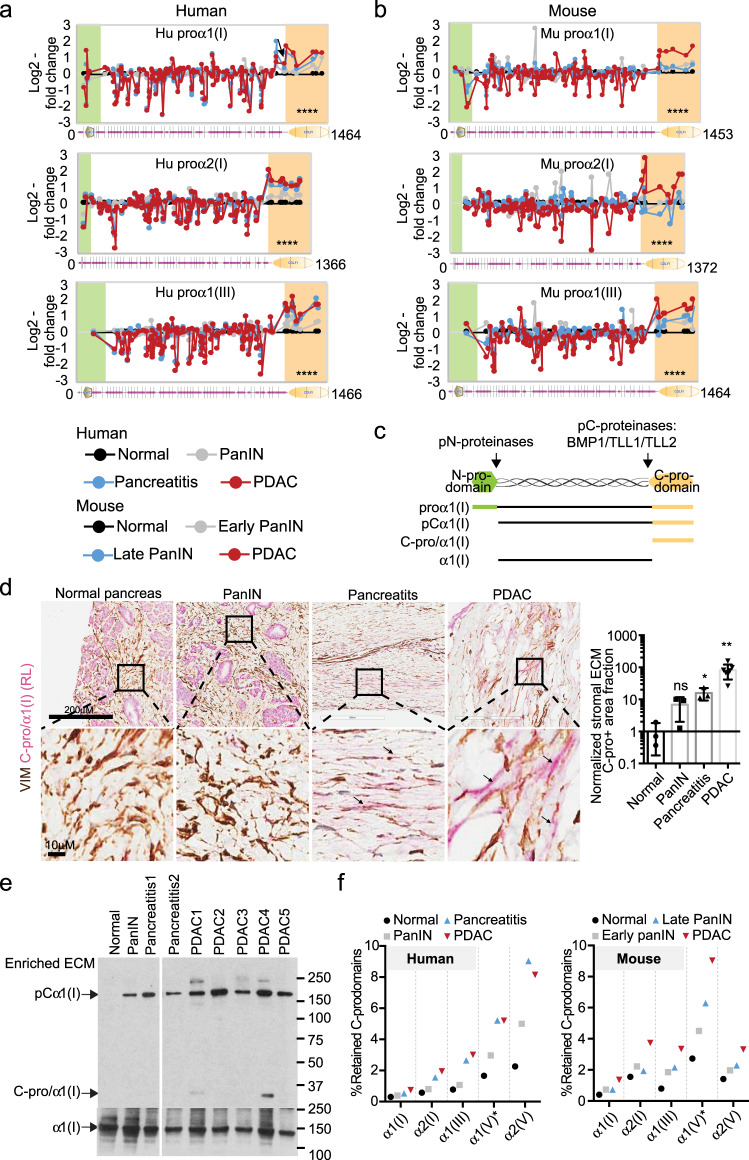
Mass spectrometry (MS) of enriched ECM from progressive PDAC stages reveals retention of fibrillar collagen C-prodomains. **a**, **b** Peptide analysis showed higher abundance of peptides located in the C-prodomains of fibrillar procollagens proα1(I), proα2(I), and proα1(III) in both human and mouse PDAC ECM compared with normal pancreas ECM. Peptides are plotted with their log2-fold change relative to normal pancreas (*y* axis) and starting amino acid location (*x* axis). Protein isoform domain structures from SMART (smart.embl-heidelberg.de) are displayed at the bottom. Green shade indicates N-prodomains and orange shade indicates C-prodomains. ****p < 0.0001. *P*-values come from two-tailed *t*-tests between the log2(fold change) in peptides from the C-prodomain region and from the mature collagen region. **c** Schematic of the processing of proα1(I) with the various forms, domains and prodomain proteinases named. **d** Double-label immunohistochemical staining showing that anti-C-pro/α1(I) staining (red, with RL antibody) is in the extracellular space (negative for the cytoplasmic marker VIM in brown) of the mesenchymal regions in PDAC and pancreatitis samples but not in normal or PanIN pancreas. The mesenchymal regions are enlarged and quantified. The quantification was done by calculating the fraction of C-pro/α1(I) positive and VIM-negative area to that of VIM-negative ECM region and then normalizing the ratios to that of normal pancreas. Arrows point to extracellular C-pro/α1(I) regions. The numbers of quantified samples are 3, 3, 3, 7 (left to right). *p < 0.05; **p < 0.01; ns not significant. Two-tailed *t*-tests are performed. All columns are represented by mean ± SD. **e** Western blotting (WB) showing large majority of the C-pro/α1(I) in the diseased pancreas ECM is present as uncleaved forms attached to α1(I), showing a representative image of three experimental repeats. **f** Estimation of the percentage of each fibrillar collagen C-prodomain that is retained in the ECM in each stage. Note that there is increased C-prodomain retention as disease progresses. The percentages were derived from the average intensity of [1] peptides that map to the C-prodomain region divided by that of [2] peptides from the mature collagen region.

We previously applied quantitative LC-MS/MS-based proteomics using tandem mass tag (TMT) labeling to characterize the enriched ECM from normal pancreas, pancreatic intraepithelial neoplasia (PanIN), chronic pancreatitis, and PDAC pancreas, and showed that many overrepresented ECM proteins in PDAC are pro-tumorigenic^11,17^. Besides protein-level changes, we focused on additional ECM changes in PDAC, such as protein domain inclusion/exclusion events. Using the TMT reporter-ion ratios, we mapped the relative abundances of individual peptides at different stages to their location along the amino to carboxy terminal (N-C) axis of fibrillar procollagens. As shown in Fig. 1 and Supplementary Fig. 1 this analysis revealed higher relative abundance of peptides from the C-prodomains of the dominant fibrillar procollagens in PDAC as compared with normal pancreas. This phenomenon was observed in the case of multiple fibrillar procollagen chains, including proα1(I), proα2(I), proα1(III), and proα2(V), and for the N-prodomain of proα1(V), and was present in both mouse and human samples (Fig. 1a, b and Supplementary Fig. 1A, B). As a negative control, peptides located to the N-prodomain region (except proα1(V), see below) were not uniformly different between PDAC and normal pancreas. The PanIN and pancreatitis samples showed intermediate retention of the C-prodomains (Fig. 1a, b and Supplementary Fig. 1A, B). Other fibrillar procollagens, such as procollagen II and XI, were not abundant and not covered by sufficient numbers of peptides in the prodomains to make useful comparisons. We also noted that some peptides in the collagen triple-helical regions appeared to be detected at reduced levels (Fig. 1a, b, see Discussion).

C-prodomains bring together nascent procollagen chains to form the triple-helical domain flanked by N- and C-prodomains. Subsequently, pC-proteinase activity removes C-prodomains from fully folded procollagen to enable assembly into fibrils^18^ (Fig. 1c). Unlike other fibrillar collagens, the N-prodomain of α1(V) is removed by one of the pC-proteinases^19^. As noted, we observed increased retention of the N-prodomain instead of the C-prodomain of proα1(V).

To confirm these results, we used double-label immunohistochemistry (IHC) to examine C-pro/α1(I) localization in the collagen-rich mesenchymal region of human normal pancreas, PanIN, pancreatitis samples, and PDAC tumors (Fig. 1d and Supplementary Fig. 1C). In this assay, C-pro/α1(I)-positive VIM-negative (red but not brown) signal indicated the extracellular C-pro/α1(I) signal, whereas intracellular C-pro/α1(I) staining was excluded from analysis by the VIM staining. We observed that the PDAC stromal region had a much higher level of extracellular C-pro/α1(I) and that PanIN and pancreatitis samples both have increased extracellular C-pro/α1(I) as compared with normal pancreas (Fig. 1d and Supplementary Fig. 1C).

The observed association of C-pro/α1(I) with the PDAC ECM could be due to: [1] uncleaved proα1(I) or pCα1(I), or [2] cleaved C-pro/α1(I) remaining associated with the ECM through physical binding. To distinguish these two possibilities, we performed western blotting on the enriched ECM samples, which showed that most of the C-pro/α1(I) signal came from uncleaved pCα1(I) (Fig. 1e). We also demonstrated that a peptide spanning the cleavage site had similar abundance to the other peptides mapped to the C-pro/α1(I) region in the ECM MS study (Fig. S1D). Both results suggested that there is reduced pC-proteinase activity in the PDAC ECM.

Next we estimated that 1–10% of the various C-prodomains are retained and the retention is increased ~2- to 4-fold comparing PDAC to normal pancreas across different procollagens (Fig. 1f). We have previously shown that both cancer cells and stromal cells deposit collagens^9^. Based on xenograft tumor ECM MS data^18^ (in which peptides with human sequences define cancer-cell origin) we estimated that cancer cells deposit 2–3% of the major fibrillar collagens (Supplementary Fig. 1E). Furthermore, cancer cells showed a higher percentage of retained type III collagen C-prodomain than did stromal cells (14% vs. 3%, Supplementary Fig. 1F). Hence, we next sought to understand the consequence of the missed cleavage event by directly studying the pC-proteinases in cancer cells.

### BMP1 suppresses PDAC tumor progression and metastasis

**Fig. 2:**
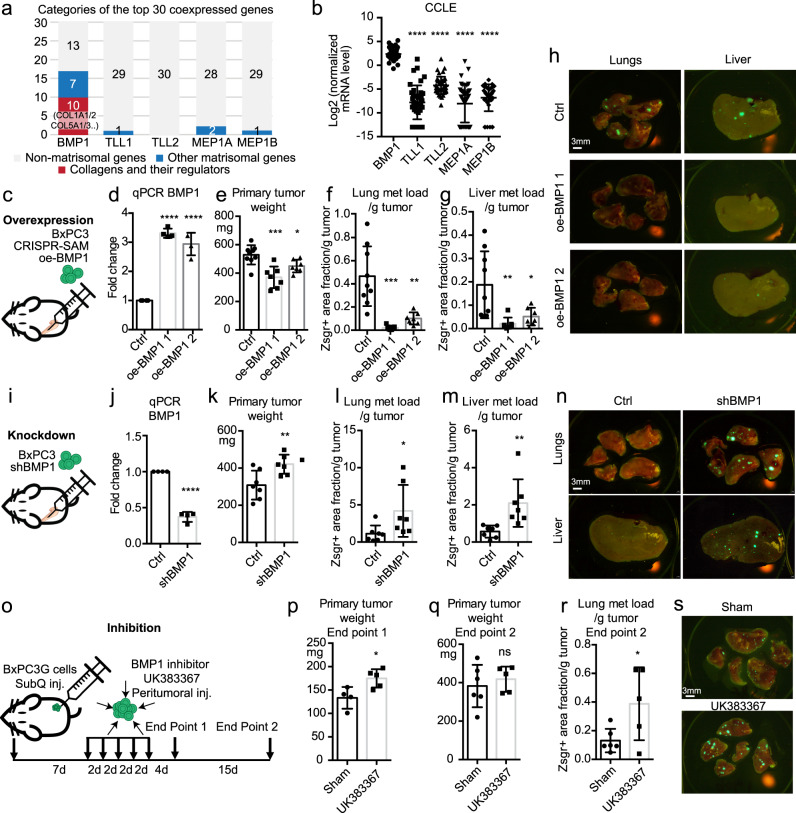
BMP1 suppresses tumor growth and metastasis in PDAC. **a** The top 30 positively coexpressed genes ranked by correlation coefficients with BMP1, but not TLL1/2, or MEP1A/1B, have a high fraction of matrisomal (blue or red) genes, especially collagen-related genes (red), using the TCGA dataset in cBioPortal. **b** BMP1 has the highest mRNA expression level in a panel of 41 cultured PDAC cell lines using the CCLE dataset. **c**–**h** Orthotopic injection of BxPC3 cells (**c**) overexpressing BMP1 with two different guides using a CRISPR-activation method (**d**) resulted in reduced primary tumor weight (**e**), and reduced lung (**f**) and liver (**g**) metastasis load after normalization to primary tumor weight. Representative lung and liver metastasis images are shown in h. Mouse numbers are 9, 7, 7 (**e**–**g**, left to right). **i**–**n** Orthotopic injection of BxPC3 cells (**i**) knocked down for BMP1 expression (**j**) resulted in increased primary tumor weight (**k**), and increased lung (**l**) and liver (**m**) metastasis load after normalization to primary tumor weight. Representative lung and liver metastasis images are shown in **n**. The numbers of mice are 7 for both groups. **o**–**s** subcutaneous injection of lung-metastasis-selected BxPC3 cells followed by peritumoral injection of BMP1 inhibitor UK383367 (**o**) resulted in increased primary tumor growth (end point 1, **p**) and increased normalized metastasis load despite no significant change in primary tumor weight (end point 2 **q**, **r**). Lung metastasis images are shown in **s**. Mouse numbers are 4 and 5 (end point 1) and 6 and 5 (end point 2, left to right). *p < 0.05; **p < 0.01; ***p < 0.001; ****p < 0.0001. ns not significant. This labeling scheme applies to all related figures. All *p*-values come from two-tailed Student’s *t*-tests. All columns are represented by mean ± SD.

pC-proteinases include astacin metalloprotease members BMP1 (bone morphogenetic protein 1), TLL (Tolloid-like protein) 1 and 2, which specifically cleave C-prodomains^20^, and Meprin α and β, which cleave both N- and C-prodomains^21^. Meprins are less likely to be responsible because we only observed altered C-prodomain retention. We surveyed the coexpressed genes in the pancreatic cancer TCGA dataset and found that BMP1 was coexpressed with a large number of matrisome genes and especially collagen-related genes, such as COL1A1 and COL1A2 (Fig. 2a and Supplementary Data 1). This was not the case for TLLs or Merpins. We also found that, in the CCLE (cancer cell encyclopedia, https://portals.broadinstitute.org/ccle) RNAseq dataset, PDAC cell lines have significantly higher expression of BMP1 than TLLs and Meprins (Fig. 2b). We therefore inferred that BMP1 is likely the most relevant pC-proteinase in PDAC.

To investigate BMP1, we used the synergistic activation mediator (SAM) CRISPR/dCas9 gene activation system^22^ to induce overexpression (oe) of BMP1 in BxPC3 cells (Fig. 2c, d). The BMP1 gene has two major transcripts, BMP1-1 and BMP1-3 (also named mTolloid, Supplementary Fig. 2A), which both encode active enzymes that cleave fibrillar procollagens^23^. The expression of both transcripts was enhanced by oe-BMP1 (Supplementary Fig. 2B). Orthotopic injection of oe-BMP1 BxPC3 cells led to a decrease in primary tumor weight (Fig. 2c, e) and a marked reduction in normalized lung and liver metastasis load (Fig. 2f–h). Oe-BMP1 also led to significant reduction in lung metastasis using an experimental tail-vein metastasis model (Supplementary Fig. 2C).

Meanwhile, orthotopic injection of BxPC3 cells knocked down for BMP1 expression by shBMP1 (Fig. 2i, j) resulted in slightly increased tumor weight (Fig. 2k), and lung and liver metastasis loads (Fig. 2l–n). Furthermore, shBMP1 in BxPC3 cells led to a significant increase in lung metastasis by tail-vein injection (Supplementary Fig. 2D). We also used an inhibitor of BMP1, UK383367, to inhibit BMP1 activity in vivo^21,22^. UK383367 resulted in an increased amount of C-pro/α1(I) on collagen fibers by immunostaining and Western blotting (WB) from cultured CAFs (Supplementary Fig. 2E–I) and decreased total collagen I deposition by WB in a dose-dependent manner in cultured CAFs (Supplementary Fig. 2G–I) and cancer cells (Supplementary Fig. 2J), confirming that it is an inhibitor of BMP1. Because of the high serum binding and turnover rate of UK383367^24,25^, we employed a subcutaneous (subQ) cancer cell injection model followed by peritumoral dosing (Fig. 2o). We injected a lung-selected more metastatic BxPC3 cell line^17^ and showed that UK383367-treated tumors have increased primary tumor weight (Fig. 2p) and lung metastasis (Fig. 2q–s). UK383367 dosing resulted in higher proliferation by Ki67 staining and no change in apoptosis by cleaved caspase 3 staining in tumors (Supplementary Fig. 2K). Combining the results from both overexpression (Fig. 2c–h) and knockdown/inhibition (Fig. 2i–s) experiments, we conclude that BMP1 suppresses tumor growth and metastasis in the BxPC3 cells.

### BMP1 promotes fibrillar collagen deposition

**Fig. 3:**
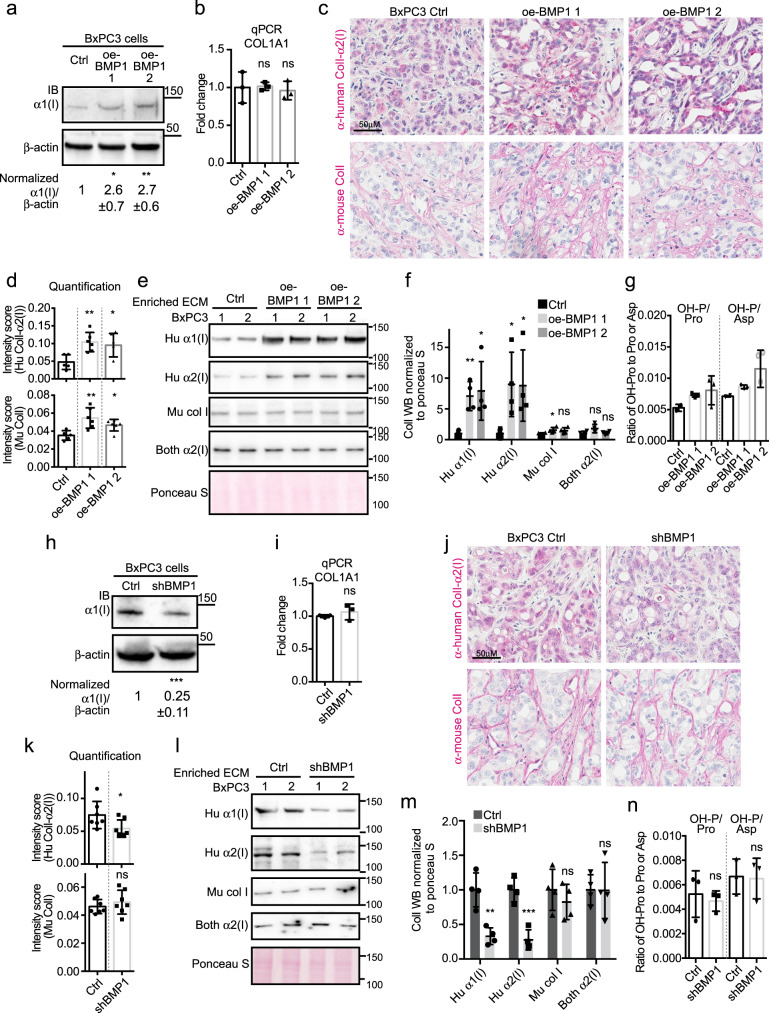
BMP1 promotes collagen I deposition. **a**, **b** Cultured BMP1 oe cells deposit more ColI compared with control BxPC3 cells (**a**) although COL1A1 mRNA levels are comparable (**b**). **a** Shows images representative of three independent experiments with β-actin as a sample processing control. The quantified WB signal is represented as mean ± SD. Note that, while α1(I) protein is elevated, mRNA is not. **c** Representative IHC on xenograft tumor sections showed increased intensities of both human cancer-cell- and murine stroma-derived ColI staining upon BMP1 overexpression. The intensities were quantified in **d**. *N* numbers are 5, 6, 6 (left to right). **e**, **f** Western blotting (WB) of enriched ECM samples from xenograft tumors showed increased cancer-cell-derived (human) ColI deposition in oe-BMP1 tumors. In **f**, each column represents the quantification of the band intensities for four tumors (two were shown in **e** and two came from an independent repeat, see Methods section). **g** The intensity of protein hydroxyproline (Hypro) normalized to either the intensity of protein proline or aspartic acid in xenograft tumors suggested a slight increase in overall collagen upon BMP1 overexpression for one of the guides but not the other. **h**, **i** Cultured shBMP1 cells deposit less α1(I) compared to control BxPC3 cells (**h**) although COL1A1 mRNA level is comparable (**i**, *N* = 3). **h** is an image representative of three independent experiments with β-actin as a sample processing control. **j** IHC on xenograft shBMP1 tumor sections showed somewhat reduced ColI staining for human cancer-cell-derived ColI but no change in stroma-derived ColI staining upon knocking down COL1A1 in cancer cells. The intensities were quantified in **k** (*N* = 7 for both columns). **l** WB of enriched ECM samples from xenograft shBMP1 tumors showed reduced cancer-cell-derived (human) ColI deposition. The WB band intensities were quantified in **m** (*N* = 4). **n** The intensity of protein Hypro normalized to either the intensity of protein proline or aspartic acid in xenograft tumors suggested no change in overall collagen quantity upon BMP1 knockdown (*N* = 3). All *p*-values come from two-tailed Student’s *t* tests. All columns are represented by mean ± SD.

Since BMP1 cleavage of C-prodomains is rate-limiting for collagen fibrillogenesis in vitro, we wished to investigate whether BMP1 promotes fibrillar collagen deposition in PDAC tumors. In cultured BxPC3 cell lysates, we observed that the level of collagen I protein was increased by BMP1 overexpression, while COL1A1 mRNA levels were not affected (Fig. 3a, b).

To distinguish cancer-cell- (human) vs/stromal-cell- (mouse) origins of collagen I (ColI) in xenograft tumors, we tested several anti-ColI antibodies both by WB and by IHC, from which we identified two (predominantly) human-specific, one murine-specific anti-ColI antibody and one antibody that recognizes both species (Supplementary Fig. 3A, B). The sequences of mature human and mouse BMP1 are 98% identical, thus we expected that human BMP1 functions on both human and mouse targets.

IHC experiments showed that oe-BMP1 in cancer cells induced higher ColI protein deposition by both cancer cells and stromal cells in the xenograft tumors (Fig. 3c, d). WB analysis on the enriched insoluble ECM showed that ECM from oe-BMP1 tumors contained a higher deposition of cancer-cell-derived ColI, while the fractions of the stromal- and cancer cell/stroma-derived ColI were only marginally increased (Fig. 3e, f). The antibody that recognizes both species gave results similar to the mouse-specific antibody, which was expected because stromal cells deposit the vast majority (98%) of all ColI (Supplementary Fig. 1E). The WB assay could not estimate stromal or total collagen changes because a major portion of total ECM (against which we normalized) is stroma-derived fibrillar collagen (~60–90%). To estimate the total collagen change, we performed GC-MS analysis on hydroxyproline (Hypro) abundance derived from protein hydrolysates from xenograft tumors as a readout for collagen abundance, since collagen is rich in hydroxyproline (Fig. 3g). We showed that oe-BMP1 led to a slight increase in total Hypro level, suggestive of an increase in overall collagen.

We then applied the same ColI quantification methods to shBMP1 xenograft tumors and showed that shBMP1 in cancer cells [1] reduced ColI protein but not mRNA in cultured cells (Fig. 3h, i); and [2] also decreased cancer-cell-derived but not stroma-derived nor overall ColI in xenograft tumors (Fig. 3j–n).

Examination of the collagen fibril formation in vivo in oe-BMP1 tumors by transmission electron microscopy (TEM) showed that oe-BMP1 tumors had smaller collagen fibril diameters (Supplementary Fig. 3C–E).

In summary, BMP1 in cancer cells drives ColI deposition, more so from cancer cells than from stromal cells, possibly because BMP1, although a secreted proteinase, can also function intracellularly and thereby affects most strongly the collagen produced by the tumor cells overexpressing it.

### Differential effects of BMP1 in PDAC cell lines with different collagen expression levels

**Fig. 4:**
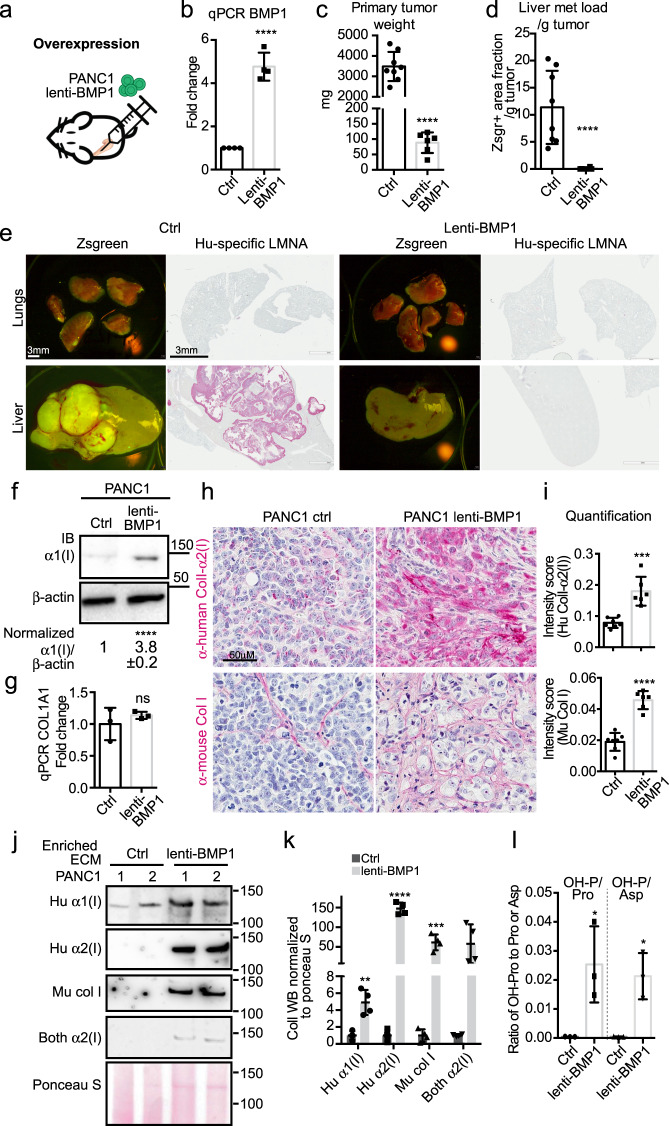
Overexpressing BMP1 in PANC1 cells suppresses tumor growth and metastasis. **a**–**e** Orthotopic injection of PANC1 cells (**a**) overexpressing BMP1 with lenti-BMP1 (**b**) resulted in reduced primary tumor weight (**c**), and reduced liver metastasis load after normalization to primary tumor weight (**d**). Representative lung and liver metastasis images are shown in **e** with both zsgreen signal and human LMNA IHC to highlight cancer cells. Note that PANC1 cells are not metastatic to the lungs. Mouse numbers are 8 and 6 (left to right). **f**, **g** Cultured lenti-BMP1 PANC1 cells deposit 4x more α1(I) compared to control cells (**f**) although COL1A1 RNA level is comparable (**g**). **f** Shows images representative of three independent experiments with β-actin as a sample processing control. The quantified WB signal is represented as mean ± SD. **h**, **i** IHC on xenograft tumor sections showed increased intensities in both cancer-cell- and stromal-derived ColI staining upon BMP1 overexpression in the PANC1 cells (**h**). The intensities were quantified in **i** (*N* = 7 and 6, left to right). **j**, **k** WB on enriched ECM samples from xenograft PANC1 tumors showed increased cancer-cell-derived (human) and stromal-derived (mouse) ColI deposition in lenti-BMP1 tumors. See Methods section for antibodies used. **k** is the quantification of the band intensities of four tumors (two were shown in **j** and two came from an independent repeat). **l** The normalized intensity of protein Hypro to either protein proline or aspartic acid in xenograft tumors suggested an increase in overall collagen upon BMP1 overexpression in PANC1 cells (*N* = 3). All *p*-values come from two-tailed Student’s *t* tests. All columns are represented by mean ± SD.

To investigate the function of BMP1 in other PDAC cells, we first lentivirally overexpressed BMP1 (BMP1-1-3, see Supplementary Fig. 2) in PANC1 cells (Fig. 4a, b). Orthotopic injections of lenti-BMP1 PANC1 cells exhibited major reductions in both primary tumor weight (Fig. 4c) and liver metastasis load (Fig. 4d, e). Lenti-BMP1 PANC1 cells displayed increased ColI protein levels but not mRNA levels (Fig. 4f, g). Lenti-BMP1 PANC1 orthotopic xenograft tumors had a markedly increased ColI deposition in both cancer-cell and stromal-cell compartments by IHC (Fig. 4h, i), by WB analysis of enriched ECM (Fig. 4j, k), and by Hypro measurement of overall collagen level (Fig. 4l). Thus, BMP1 in PANC1 cells caused increased collagen deposition and reduced tumor growth and metastasis to a greater extent than was observed with BxPC3 cells (Fig. 3).

In contrast, CRISPR-SAM oe-BMP1 in AsPC1 cells did not alter the orthotopic xenograft primary tumor weight or lung metastasis (Supplementary Fig. 4A–E) and did not change the ColI protein level in cultured cells (Supplementary Fig. 4F, G), nor in the xenograft tumors by IHC of tumor sections (Supplementary Fig. 4H, I), WB of enriched ECMs (Supplementary Fig. 4J, K) or Hypro measurement of collagen levels (Supplementary Fig. 4L). We confirmed that in the AsPC1 xenograft tumors BMP1 is overexpressed at both the protein level by IHC (Supplementary Fig. 4M) and at the mRNA level by qPCR with validated human-specific primers (Supplementary Fig. 4N–Q).

The differential effect of BMP1 in BxPC3, PANC1, and AsPC1 cells suggested cell-intrinsic differences. Among them, PANC1 cells had the highest COL1A1 expression and the largest tumor-suppressing effect by BMP1, while AsPC1 had the lowest COL1A1 expression and no BMP1 effect (see the next section for COL1A1 expression data). Hence we investigated directly whether the ability of BMP1 to suppress growth depends on the level of fibrillar collagen in the PDAC cell lines.

### BMP1 functions through promoting ColI deposition in cancer cells to suppress tumor growth and metastasis

**Fig. 5:**
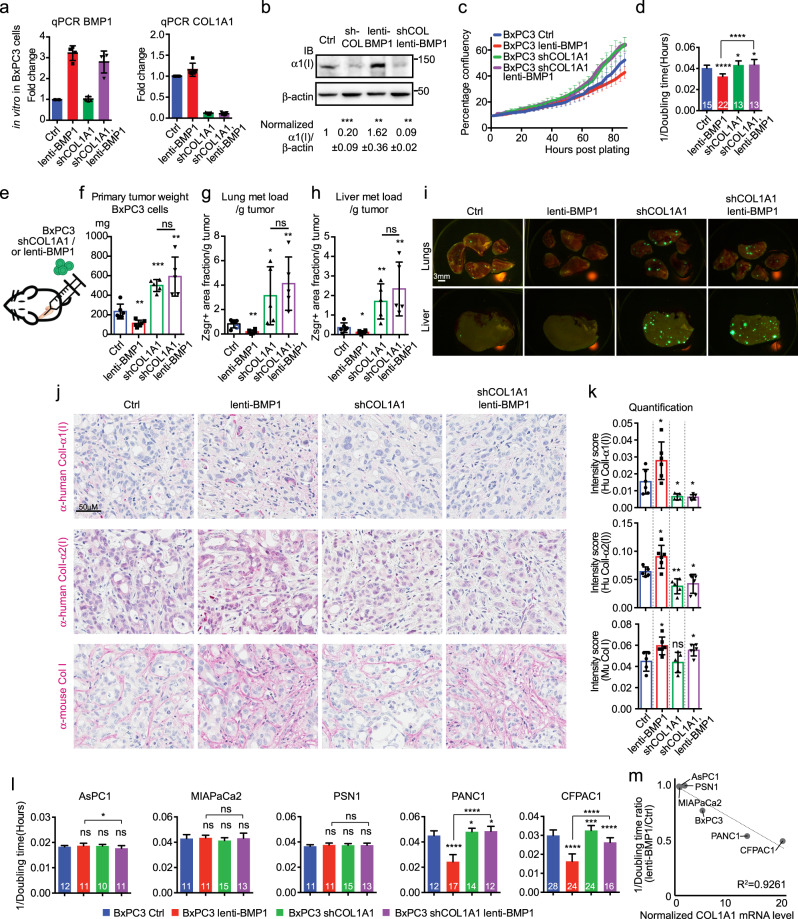
BMP1 functions through promoting collagen I deposition in cancer cells to suppress tumor growth and metastasis. **a** Generation of BxPC3 cells that either overexpress BMP1 (red) or are knocked down for COL1A1 (green) or modified for both (purple), as indicated by qPCR for BMP1 and COL1A1 (*N* = 4). **b**, In cultured BxPC3 cells, lenti-BMP1 promoted ColI deposition while shCOL1A1 reduced ColI deposition irrespective of BMP1 levels. β-actin is a sample processing control. **c**, **d** In cultured BxPC3 cells, shCOL1A1 promoted cell growth, and rescued the reduced cell growth by lenti-BMP1 to the same level as shCOL1A1 alone, as shown by growth curves (**c**) and growth rate represented by 1/doubling time (**d**). *N* numbers for **c** and **d** are indicated on the graph in white text in **d**. Color coding is as in **a**. **e**–**i** orthotopic injection of BxPC3 cells (**e**) overexpressing BMP1 using lenti-BMP1 reduced primary tumor weight (**f**) and lung and liver metastasis load (**g**, **h**), whereas shCOL1A1 in lenti- BxPC3 cells expressing BMP1 resulted in increases in [1] primary tumor weight and [2] lung and liver metastasis, to the same levels seen with shCOL1A1 alone (**f**–**h**). Representative images of lung and liver metastasis are shown in I. Mouse numbers are 6, 6, 5, 5 (left to right). **j** IHC on xenograft tumor sections showed that cancer-cell-derived ColI was increased by lenti-BMP1 but reduced by shCOL1A1, even in lenti-BMP1-expressing cells, while stroma-derived ColI was also increased by lenti-BMP1 and not changed by shCOL1A1. The intensities were quantified in **k**. *N* numbers are 6, 6, 5, 6 (left to right). **l** in AsPC1, MIAPaCa2, and PSN1 cells, shCOL1A1 and lenti-BMP1 had no effect on cell growth. However, in PANC1 and CFPAC1 cells, shCOL1A1 promoted cell growth, lenti-BMP1 reduced cell growth and that reduction was rescued by shCOL1A1. *N* numbers are indicated on the graph in white text. Color coding is as in **a**. **m** The amount of cell growth reduction by lenti-BMP1 expression in PDAC cells showed a linear correlation with their endogenous COL1A1 expression levels in the CCLE dataset normalized. All *p*-values come from two-tailed Student’s *t* tests. All columns are represented by mean ± SD.

ColI, a heterotrimer with two units of α1(I) and one unit of α2(I), is the most abundant fibrillar collagen species made by both human cancer cell lines and stromal cells in xenograft PDAC tumors (50–70% of all fibrillar collagen mass, Supplementary Fig. 5A). We therefore generated three separate cell lines from BxPC3: [1] overexpressing BMP1 by lenti-BMP1, [2] knocked down for COL1A1 expression by shCOL1A1, or [3] both overexpressing BMP1 and knocked down for COL1A1 (Fig. 5a). On WB, lenti-BMP1 enhanced α1(I) protein level, which was knocked down by shCOL1A1 (Fig. 5b). In vitro growth assay showed that [1] lenti-BMP1 cells slowed down cell growth, whereas shCOL1A1 cells grew faster, compared to control cells; [2] shCOL1A1 completely reversed the growth suppression by lenti-BMP1 (Fig. 5c, d).

We then orthotopically implanted the same group of cells in vivo (Fig. 5e). We found that the primary tumor growth and lung and liver metastasis load resembled the in vitro growth results, in which lenti-BMP1 reduced and shCOL1A1 promoted tumor growth and metastasis, and shCOL1A1 completely rescued the suppression by lenti-BMP1 (Fig. 5f, g). These results suggested that the function of BMP1 in suppressing tumor growth and metastasis requires ColI produced by the cancer cells.

Next we quantified ColI deposition in the xenograft tumors. We found that, compared to the control tumors, lenti-BMP1 tumors have more ColI deposition both by cancer cells and, to a lesser extent, stromal cells, however, lenti-BMP1/shCOL1A1 tumors have reduced ColI deposition from cancer cells, but somewhat increased stromal ColI deposition both by IHC staining (Fig. 5j, k) and by WB on enriched ECM (Supplementary Fig. 5B, C). The overall collagen level was similarly increased by BMP1 overexpression in both lenti-BMP1 and lenti-BMP1/shCOL1A1 tumors (Supplementary Fig. 5D).

We also extended this analysis to additional cell lines, including AsPC1, MIAPaCa2, PSN1, PANC1, and CFPAC1, using an in vitro growth assay (Supplementary Fig. 5E, F). We showed that PANC1 and CFPAC1 reduced cell growth rate in response to BMP1 overexpression and that was obviated by COL1A1 knockdown, however, AsPC1, MIAPaCa2, and PSN1 cells failed to respond to lenti-BMP1 or shCOL1A1 (Fig. 5l and Supplementary Fig. 5G). This panel of cells has a wide range of endogenous COL1A1 mRNA expression in CCLE RNAseq data (~20 fold); PANC1 and CFPAC1 had much higher COL1A1 expression than AsPC1, MIAPaCa2 and PSN1 cells, which we confirmed by qPCR and WB (*R*^2^ to the CCLE RNAseq data is 0.9733 and 0.7170, respectively). In fact, we found that the extent of tumor growth suppression by BMP1 correlated linearly with the endogenous COL1A1 expression levels (Fig. 5m).

Ascorbic acid (ASC) can induce collagen secretion^26^, and high-dose ASC was shown to cause cytotoxicity through oxidative stress to PDAC cells^27^. We found that ASC treatment suppressed in vitro cell growth in both AsPC1 and PANC1 cells, and that the suppression effect was partially mediated by ColI in high COL1A1-expressing PANC1 cells, because shCOL1A1 partially eliminated their growth reduction, but not in low COL1A1-expressing AsPC1 cells (Supplementary Fig. 5H, I). Altogether, we showed that BMP1 depends on cancer-cell-derived COL1A1 for its function to suppress PDAC tumor growth and metastasis.

### BMP1 most likely functions through cleavage of ColI to promote its deposition

Apart from fibrillar procollagens, BMP1 has many other targets, such as SLRP proteoglycans (decorin, biglycan, osteoglycin), LTBP1, LOX/LOXL, and Perlecan, which are implicated in multiple processes, including collagen fibrillogenesis (SLRPs, LOX/LOXL), TGF-β signaling (LTBP1), and angiogenesis (HSPG2)^28^. We performed peptide level analysis to survey BMP1’s other targets and found that HSPG2 LG3 domain is partially but significantly retained in diseased tissues (PanIN, pancreatitis, and PDAC) as compared to normal pancreas (Supplementary Fig. 6). We did not find any evidence for reduced processing of the other potential substrates listed. Because many BMP1 targets directly or indirectly impact fibrillar collagens, we next asked whether fibrillar collagens are the direct substrates of BMP1 and mediators of its anti-tumorigenic roles.

**Fig. 6:**
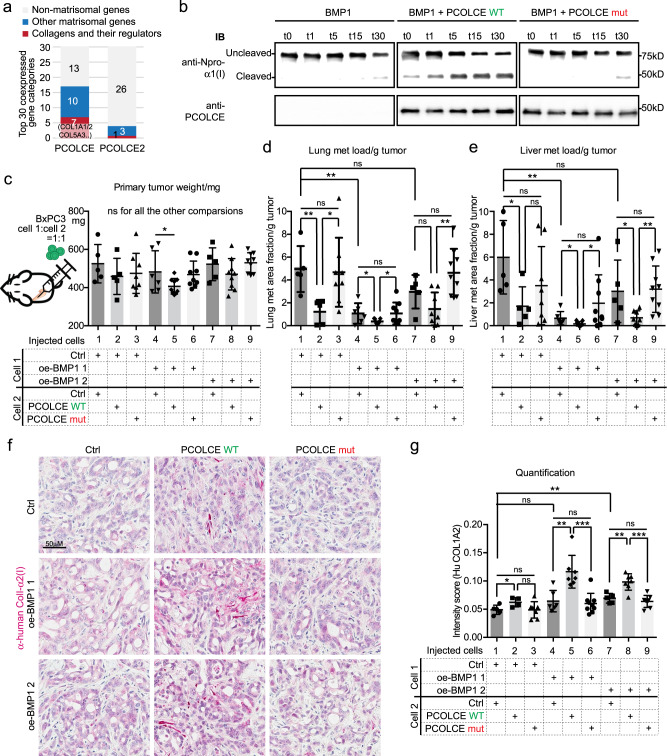
PCOLCE promotes BMP1 suppression of metastasis. **a** The top 30 positively coexpressed genes ranked by correlation coefficients with PCOLCE have a higher fraction of matrisomal (blue or red) genes, especially collagen-related genes (red), than PCOLCE2 using the TCGA dataset in cBioPortal. **b** In vitro BMP1 cleavage assay suggested that WT PCOLCE, but not mut PCOLCE, could promote BMP1 cleavage of mini-proα1(I). See Fig. S7A–D for details of the cleavage assay. Images are representative of two independent experiments. **c**–**e** Orthotopic injection experiment using 1:1 mixes of cells that overexpress BMP1 and PCOLCE (WT or mut). **c** WT PCOLCE, but not mut PCOLCE, reduced primary tumor size of one of the lines overexpressing BMP (oe-BMP1 1; significant difference between lanes 4 and 5), but not the second (no difference between lanes 7 and 8 in **c**). **d**, **e** Both BMP-oe lines expressing WT PCOLCE showed significant suppression of lung and liver metastasis (significant differences between lanes 4 and 5, and between lanes 7 and 8; images shown in Fig S7E). However, the mutant form of PCOLCE did not show the same effect (no significant difference between lanes 4 and 6, nor between lanes 7 and 9 in **d** and **e**). Mouse numbers for each combination are 5, 5, 8, 6, 9, 9, 5, 9, 9 (left to right). **f**, **g** IHC on xenograft tumor sections (**f**) and the quantification (**g**) showed that cancer-cell-derived ColI was increased by oe-BMP1 and was further increased by the expression of WT PCOLCE but not that of mut PCOLCE (significant differences between lanes 4 and 5, and between lanes 7 and 8, but no difference between lanes 4 and 6, nor between lanes 7 and 9 in **g**). *N* numbers are 5, 4, 7, 6, 7, 9, 5, 7, 8 (left to right). All *p*-values come from two-tailed Student’s *t* tests. All columns are represented by mean ± SD.

PCOLCE (Procollagen C-Endopeptidase Enhancer) and its family member PCOLCE2 (Supplementary Fig. 6A) are both BMP1 enhancers. PCOLCE, but less so PCOLCE2, is coexpressed with matrisomal and especially collagen genes in the PDAC tumors (Fig. 6a and Supplementary Data 1). PCOLCE enhances BMP1 cleavage of procollagens by directly binding to the C-prodomains^29,30^. Consequently, PCOLCE specifically stimulates the activity of BMP1 on procollagens but not other targets^31^. We therefore investigated whether PCOLCE could enhance the anti-tumorigenic role of BMP1, which, if so, could suggest that BMP1 suppresses tumor growth and metastasis through cleavage of fibrillar collagens.

We mutated the previously identified critical sites of PCOLCE function^29,30^ (Supplementary Fig. 7A). An in vitro BMP1 cleavage assay using a mini-proα1(I) construct (Supplementary Fig. 7B) showed that purified wild type (WT), but not mutated (mut) PCOLCE protein (Supplementary Fig. 7C), could promote the cleavage of mini-proα1(I) by BMP1 in vitro (Fig. 6b). We therefore generated BxPC3 cells expressing similar levels of WT and mut PCOLCE (Supplementary Fig. 7D). We mixed PCOLCE WT and mut cells with control cells at 1:1 ratio for orthotopic injection. PCOLCE WT, but not PCOLCE mut, reduced lung and liver metastasis (bars 1–3 in Fig. 6c–e and Supplementary Fig. 7E). When, instead of control cells, oe-BMP1 1 cells were mixed with PCOLCE WT cells or PCOLCE mut cells, the tumor growth and the lung and liver metastasis were reduced (lanes 4–6 in Fig. 6c–e and Supplementary Fig. 7E). Mixing PCOLCE WT cells with a second line of oe-BMP1 2 cells resulted in a similar reduction in metastasis (lanes 7–9 in Fig. 6c–e and Supplementary Fig. 7E). In both cases the further suppression of metastasis by WT but not mutant PCOLCE was retained. Quantification of cancer-cell-derived ColI level by IHC showed that WT, but not mut, PCOLCE further promoted ColI deposition in oe-BMP1 tumors (Fig. 6f, g). In summary, PCOLCE enhanced BMP1 suppression of metastasis and perhaps primary tumor growth, which supports the hypothesis that BMP1 acts via cleavage of procollagens to suppress tumor growth and metastasis.

**Fig. 7:**
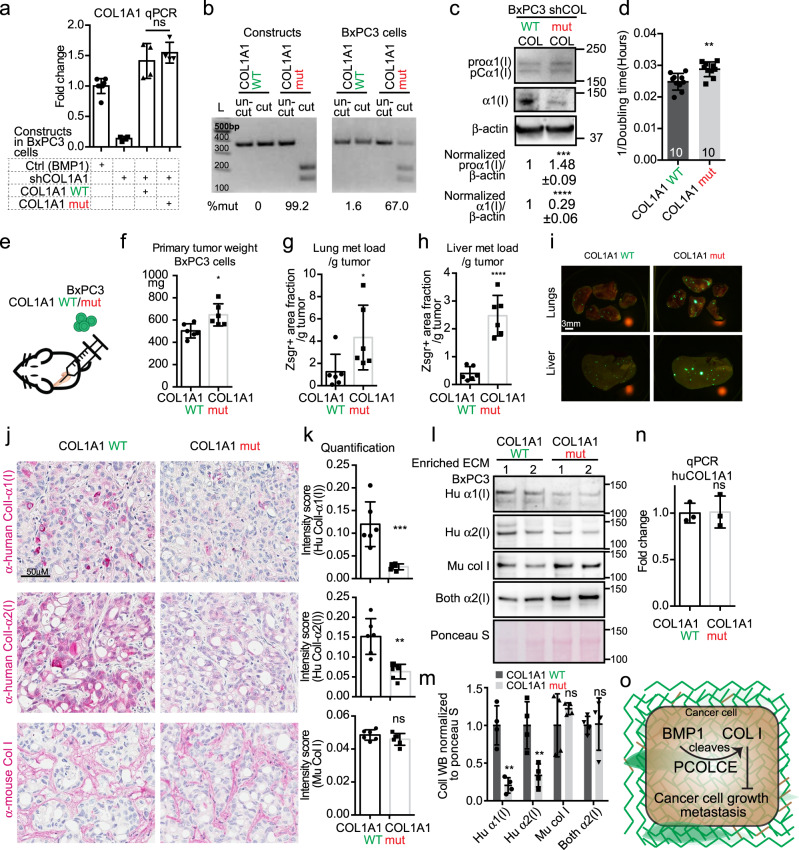
COL1A1 mutated to remove the BMP1 cleavage site promotes tumor growth and metastasis. **a** Generation of cell clones that were knocked down for COL1A1 by shRNA targeting the 5′ UTR region and expressing either WT or mutated COL1A1. qPCR showed comparable expression levels for WT and mut COL1A1 (*N* = 4). **b** Genotyping for the COL1A1 mutated transcript showed that 67% of all COL1A1 transcripts in the cells expressing COL1A1 mut were mutated (see Methods section). **c** Cultured cells expressing WT COL1A1 deposit more α1(I) compared to those expressing mut COL1A1 (A) although COL1A1 RNA levels are comparable (see panel **a**). **c** Shows images representative of three independent experiments with β-actin as a sample processing control. The quantified WB signal is represented as mean ± SD. **d** Cultured cells expressing COL1A1 mut have slightly faster growth rate than do cells expressing COL1A1, represented here by 1/doubling time in hours. **e**–**i** Orthotopic injection of BxPC3 cells expressing COL1A1 mut compared to cells expressing COL1A1 WT resulted in slightly increased primary tumor weight (**f**), and increased metastasis load in lung (**g**) and liver (**h**). Representative lung and liver metastasis images are shown in **I**. Mouse numbers are 6 for both groups. **j**, **k** IHC on xenograft tumor sections showed reduced staining intensities for cancer-cell-derived ColI but not for stroma-derived ColI staining upon BMP1 mutation. **k** is the quantification of the band intensities of four tumors from two independent experiments. **l**, **m** WB on enriched ECM samples from xenograft tumors showed decreased cancer-cell-derived (human) ColI deposition in COL1A1 mut tumors but a slight increase in mouse ColI. The WB band intensities were quantified in **m** (*N* = 4). **n** COL1A1 expression was similar in the COL1A1 WT and mut xenograft tumors in species-specific COL1A1 qPCR experiment using human-specific COL1A1 and β-actin primers (*N* = 3). **o** Working hypothesis summarizing the findings: in cancer cells, BMP1 promotes increased ColI deposition, which can be enhanced by PCOLCE and disrupted by mutating the BMP1 cleavage site in α1(I), and that, in turn, leads to suppression of tumor growth and metastasis. All *p*-values come from two-tailed Student’s *t* tests. All columns are represented by mean ± SD.

We next used lentiviral vectors to express WT COL1A1 or COL1A1 with a mutated BMP1 procollagen cleavage site (mut) in BxPC3 cells that were knocked down for endogenous COL1A1. We selected clones that expressed comparable levels of WT COL1A1 or mut COL1A1 mRNAs (Fig. 7a). We estimated the percentage of transcripts carrying the mutation by genotyping and found that the cell line expressing mut COL1A1 expressed ~67% mut COL1A1 transcript (Fig. 7b). Compared with the WT COL1A1 cells, mut COL1A1 cells showed reduced ColI protein level and slightly increased proα1(I) and pCα1(I) levels (Fig. 7c). Mut COL1A1 cells showed small but significant increases in their in vitro proliferation rate (Fig. 7d), as well as primary tumor weight (Fig. 7f) and lung and liver metastasis load (Fig. 7g–i) when orthotopically implanted in comparison to WT COL1A1 cells. Similar to what was seen in vitro, the xenograft tumors from mut COL1A1 cells deposited less cancer-cell-derived ColI by IHC (Fig. 7j, k) and by WB on enriched tumor ECM (Fig. 7l, m). The stroma-derived ColI did not appear changed by expression of mut COL1A1 (Fig. 7j–m). Human-specific qPCR indicated that the COL1A1 mRNA level from the cancer cells in the xenograft tumors was the same between mut and WT COL1A1 tumors (Fig. 7n), suggesting that the reduced ColI protein change arose post-transcriptionally, as predicted from the nature of the mutation.

Both the PCOLCE and mut COL1A1 experiments supported the notion that BMP1, with help from PCOLCE, cleaves procollagen I to stimulate its deposition, which leads to suppression of tumor growth and metastasis (Fig. 7o).

## Discussion

Fibrillar collagens are the most prominent components of the PDAC ECM and have many pro-tumorigenic roles. Here we discovered the persistence of partially uncleaved C-prodomains in fibrillar collagens that likely resulted from insufficient BMP1 activity in PDAC. This led to the further discovery that BMP1 and its cancer-cell-derived fibrillar procollagen substrates both act as suppressors of PDAC tumor growth and metastasis. Our study thus uncovered an unexpected tumor-suppressing function of cancer-cell-derived fibrillar collagens that is independent of effects on stromally derived fibrillar collagen.

Attenuated BMP1 function in humans leads to osteogenesis imperfecta (OI), a disease involving impaired fibrillogenesis of collagen, which otherwise is frequently caused by dominant mutations in α1(I) or α2(I)^32–34^. Analyses of BMP1 functions in cancer are somewhat limited and conflicting. High BMP1 levels predict poor survival in cancers such as renal clear-cell carcinoma (ccRCC)^35^ but predict better survival in breast cancer^36^. BMP1 enhances malignancy of hepatocellular carcinoma^37^ and ccRCC^35^, promotes cancer cell motility in gastric cancer^38^, but induces cellular quiescence in prostate cancer cells^39^. Here, we showed that BMP1 can suppress tumor growth and metastasis in PDAC models by promoting fibrillar collagen deposition, but only in cancer cells expressing substantial amounts of collagen. Whether the different roles of BMP1 in other cancers relate to the amount of collagen produced will be an interesting question for future studies.

The cause of the insufficiency in BMP1 activity in PDAC could be multifactorial. For example, although BMP1 is usually upregulated with collagens in cancers, including PDAC^11,35,36^, the upregulation of BMP1 expression may not be sufficient to effectively cleave the greatly elevated levels of fibrillar collagens. Thus, BMP1 may be rate-limiting for collagen fibrillogenesis in these tumors. Post-translational modifications may also impact BMP1 activity; for instance, N-linked glycosylation regulates its activity and secretion^40^.

While it is known that complete removal of the C-prodomains is required for efficient fibrillogenesis in vitro^41^, the in vivo situation may be different because of the many binding partners involved in fibril formation. Our results suggest that collagen fibrillogenesis can still happen with the inclusion of a small percentage of procollagens or pCcollagens (1–2% in Fig. 1f). We also directly observed that the C-pro/α1(I) localized to the mature collagen fibers in cultured fibroblasts, especially when BMP1 is inhibited (Supplementary Fig. 2C). In conformity with that observation, collagen fibers still form with some morphological differences in Bmp1 and Tll1 double null embryos^42^ and in human patients having COL1A1 and COL1A2 C-prodomain cleavage site mutations^43^.

This possible incorporation of procollagens into collagen fibrils may have downstream consequences, for example, collagen fibril crosslinking, solubility, protein binding, and signaling to cells may be affected. Concordantly, fibrillar collagens in murine KTC PDAC tumors are more soluble than those in normal pancreas^10^. Our TEM results on oe-BMP1 tumors revealed reduced collagen fibril diameters upon BMP1 overexpression (Supplementary Fig. 3C–E). This is consistent with previous findings, in which collagen fibril diameters in tendon and dermis were increased in mice postnatally knocked out for Bmp1 and Tll1^44^ and in human patients with BMP1 biallelic loss-of-function mutations^34^. The functional consequences of the altered fibril diameter need further study.

Our study uncovered an unexpected anti-tumorigenic role of cancer-cell-derived ColI in PDAC, a property not shared by stroma-derived ColI. One key message of our study is that cancer-cell-derived and stromal-cell-derived fibrillar collagens may have different effects on tumors. The roles of fibrillar collagens in cancer have been studied mostly with reference to the bulk of stromal collagens (mostly derived from stromal cells such as CAFs) and these have been reported to play predominantly tumor-promoting roles in cancer progression^45,46^. In contrast, very few studies have focused on in vivo cancer-cell-derived fibrillar collagens. Our study not only showed functions of cancer-cell-derived ColI in reducing tumor growth and metastasis, but also suggested that cancer-cell-derived fibrillar collagens appear to function independently of stromal-cell-derived collagens to *suppress* cancer cell growth and metastasis. Unlike control cells, cancer cells knocked down for COL1A1 no longer respond to the increase in stromal-cell-derived collagen caused by lenti-BMP1. Thus cancer-cell-derived ColI is critical for BMP1’s effects on tumor progression, whereas stromal-cell-derived ColI is either less, or not, important in this particular role, despite its prevalence.

At this point, one can only speculate as to how cancer-cell-derived ColI can function independently of stromal-cell-derived ColI to impact tumor growth and metastasis. It is unlikely that cancer-cell-made ColI functions through bulk contributions to the overall collagen-rich matrix, because [1] cancer cells contribute only 2–3% of total fibrillar collagens; [2] an increase in the stromal collagen itself is not sufficient. Thus, it appears that cancer-cell-derived ColI has some qualitatively different effects distinct from those of stromal-cell-derived ColI. Some possible mechanisms could include; [1] local deposition of cancer-cell-derived collagens to selectively modify the environment around cancer cells. Cells are known to deposit fibrillar collagens via fibripositors, structures that assemble and deposit assembled collagen fibrils adjacent to the cells that synthesize the collagen^18,47^. Such local deposition could have mechanical or biochemical effects on cancer cells, not observed with stromal-cell-derived collagens deposited further away. For instance, the cancer-cell-derived procollagens or nascent collagen fibrils may signal differentially to suppress cancer cells. [2] Since various post-translational modifications and collagen assembly into triple-helical protomers and further assembly into fibrils all occur intracellularly, those processes may differ between cancer cells and stromal cells, thereby conferring different functions on the cancer-cell-derived collagens. As mentioned in the results section, we also noted that peptides in the collagenous triple-helical regions are underrepresented (Fig. 1 and unpublished data). That could be due to various post-translational modifications, such as glycosylation and citrullination, which could differ between cancer and stromal cells and might confer different properties. Those could include differing associations with additional components such as growth factors and other soluble components; chaperones, collagen-modifying enzymes, including hydroxylases, crosslinkers, or other associated matrisomal components. Furthermore, such associated proteins could become associated with the collagen fibrils either during passage through the secretory pathway (and thus be specific to the cancer cells) or could be differentially bound by the fibrils extracellularly but be differentially affected by qualitative differences in the cancer-cell-derived fibrillar collagens, as discussed above. Any such associated proteins could have differential effects on tumor progression. [3] Unlike normal fibroblasts, which produce exclusively α1_2_α2, cancer cells produce a fraction (15–50%) of type I collagen homotrimers α1_3,_ which has been shown to promote proliferation and migration of cancer cells^48,49^. Using data from Supplementary Fig. 1E and assuming all stroma-derived ColI is heterotrimers, we estimated that, on average, 20% of cancer-cell-derived ColI is homotrimer α1_3_. The tumor growth suppression role of BMP1 and cancer-cell-derived ColI may be due to the ratio imbalance of the ColI homotrimer and heterotrimer, which may have differential impacts on cancer cells, induced by BMP1 manipulation. Overall, this is an area requiring future investigation.

The roles of BMP1 and cancer-cell-derived fibrillar collagens in suppressing tumor growth and metastasis reported here and the well accepted pro-tumorigenic roles of stromal-cell-derived fibrillar collagen clearly illustrate the functional complexity of fibrillar collagens in PDAC. This study suggests that tumor epithelial BMP1/COL1A1 expression may be a positive prognostic factor, although bulk fibrillar collagen level was shown to correlate negatively with patient survival^50^. The bulk fibrillar collagen has also been shown to trap T cells and prevent T cells from contacting tumor cells^51^, which likely contributes to the lack of efficacy in immune checkpoint blockade therapies in PDAC. Thus when considering pan-tumoral inhibition of fibrillar collagen deposition as a combination therapeutic option in PDAC, assessing the tumor epithelial fibrillar collagen expression could be necessary and effective. If such collagen inhibition strategy involves BMP1 targeting, the various effects on BMP1’s other targets need to be evaluated, for example, the lesser processing of LG3 domain of HSPG2 enhances angiogenesis^52^.

PDAC treatments could also alter fibrillar collagen and/or BMP1 expression. Upregulation of many fibrillar collagens, including COL1A2, is a response to gemcitabine treatment in PDAC cancer cells (by analyzing data in ref. ^53^). High-dose ascorbic acid (ASC) treatment combined with gemcitabine/Abraxane is under a phase II trial (NCT02905578), due to preclinical studies in which high-dose ASC induced cytotoxicity and oxidative stress selectively on PDAC cells but not normal cells^27^. ASC is also known to stimulate collagen secretion^26^. Our results suggested that the growth suppression response to sublethal concentrations of ASC may be partially through enhancing ColI secretion and fibrillogenesis. Thus patients stratified by high tumor epithelial ColI may benefit more from gemcitabine and high-dose ASC treatment. When selecting genetically modified or syngeneic transplant mouse models for preclinical tests regulating or targeting fibrillar collagens, models with a range of cancer-cell-derived COL1A1 expression need to be assessed to be representative of the wide variations seen in human PDAC.

Our previous PDAC ECM proteomic study suggested that stromal-cell-derived matrisome proteins can be both pro- and anti-tumorigenic^11^. General ECM depletion by targeting SHH signaling led to adverse effects in patients^9^. A phase III clinical trial using PEGPH20 to target hyaluronan also was discontinued because of no increase in overall or progression-free survival^54^. These results suggest that targeting aspects of the desmoplastic ECM of PDAC, while possibly important as a combination treatment, is not a simple task; it requires precise and possibly subtle change of the tumor ECM, for example, normalization or targeted depletion of certain subsets of the CAFs may be a more promising treatment option^55^. Our study presents an example of another layer of complexity when considering ECM targeting—differential responses to fibrillar collagens derived from epithelial or stromal cells and another way of stratifying patients—high vs. low levels of COL1A1 expressed by epithelial cells may lead to different responses to treatments that alter fibrillar collagen expression.

Lastly, cancer-cell-derived fibrillar collagen expression may offer an attractive therapeutic agent delivery target. Antibodies against an alternatively spliced EIIIB domain of fibronectin have been successfully developed as a highly tumor-specific targeting agent for imaging and therapy^56–58^. Similarly, the C-prodomains of fibrillar collagens may serve as another group of targets for potential PDAC early diagnosis and therapeutic agent delivery.

## Methods

### Cell line maintenance and mouse strains

The MIT Animal Care and Use Committees reviewed and approved all animal studies and procedures. NOD/SCID/IL2Rγ-null mice (Jackson Laboratory) were used throughout the study. Mice were housed on a 12-h light/12-h dark cycle at ~21 °C and 40–60% humidity. The human pancreatic adenocarcinoma (PDAC) cell lines AsPC1, BxPC3, PANC1, and MIAPaCa2 and HEK 293FT cells were purchased from American Type Cell Culture (ATCC), where they were tested and authenticated; PSN1 and CFPAC1 were gifts from the Koch Institute cell line repository, and they were originally tested and authenticated from ATCC. The human CAF cell line hT1 and hM1 were published previously^59^. All cells were cultured in media supplemented with 10% fetal bovine serum (FBS, Invitrogen) at 37 °C in a 5% CO2 incubator. AsPC1 was cultured in RPMI medium 1640 (ThermoFisher); 293FT, BxPC3, PANC1, MIAPaCa2 and PSN1 were cultured in Dulbecco’s modified Eagle’s medium (DMEM, ThermoFisher); CFPAC1 was cultured in Iscove’s modified Dulbecco’s Medium (ThermoFisher).

### Immunohistochemistry

IHC was done as previously described^60^. Details are shown in Supplemental information. Primary antibodies used were: C-pro/α1(I) (600-401-D19, RL, Rockland, 1:200);: C-pro/α1(I) (LF42, Kerafast, 1:4000); VIM (ab92547, Abcam, 1:2000); lamin A/C (ab108595, Abcam, 1:2500); human ColI/α1(I) (AF6220, R&D systems, 1:75); human ColI/α2(I) (A5786, Abclonal, 1:500); mouse ColI (AB765P, Millepore, 1:100); BMP1 (ab118520, Abcam, 1:250); Ki67 (SP6, VALENT, 1:50); and Cleaved caspase 3 (5A1E, Cell Signaling, 1:800).

### Western blotting and quantification

The ECM enrichment method was the same as previously published^11^. In brief, we used the CNMCS compartment protein extraction kit (Millipore) to decellularize tissue samples ranging from 50 mg to 100 mg. Frozen samples were homogenized with a Bullet Blender (Next Advance) according to manufacturer’s instructions. The lysates were incubated in a series of buffers to remove sequentially; (1) cytosolic proteins; (2) nuclear proteins; (3) membrane proteins; and (4) cytoskeletal proteins. One modification from previous protocols is that we used 20% CS + 80% M buffer instead of 100% CS buffer for removal of cytoskeletal proteins for better retention of ECM proteins. The remaining insoluble pellet was the enriched ECM used for immunoblotting. Enriched tumor ECM samples were lysed in 2x Laemmli buffer with 5% β-mercaptoethanol (β-ME, 1610737, Biorad). Cells were scraped off plates in 2x Laemmli buffer with 5% β-ME. Proteins were separated by SDS-PAGE (4–20% gradient gel from Biorad) and transferred to nitrocellulose membranes. For cell lysates blotting, 15 μl sample volume and large molecular weight transfer protocol (Biorad) were used to detect collagens, whereas 5 μl samples were loaded for β-actin as sample processing controls. The blots were then ponceau S stained, immunoblotted, and imaged with a Tanon 5200CE Chemi-Image System (Tanon). The following antibodies were used: C-pro/α1(I) (LF42, Kerafast, 1:1000); β-actin (14-4, Hynes lab generated, 1:5000); human α1(I) (AF6220, R&D systems, 1:500); human α2(I) (A5786, Abclonal, 1:2000); mouse ColI (AB765P, Millepore, 1:1000); α2(I) (A16699, Abclonal, 1:2000); GAPDH (MAB374, Millipore, 1:5000); N-pro/α1(I) (LF39, Kerafast, 1:2000); and PCOLCE (A15298, Abclonal, 1:2000). Uncropped images were shown in supplemental information.

Densitometry analysis was performed in ImageJ using “Mean gray value” measurements taken on bands and ponceau S staining regions (100 kD and up). The signal of the bands was normalized to ponceau S signal. Exposures yielding signals within the linear range were quantified.

### CRISPR activation

BxPC3 and AsPC1 cells stably expressing dCas9-VP64-Blast (Addgene #61425) and MS2-P65-Hygro (Addgene #61426) were generated through sequential lentiviral transduction and selection with Blasticidin and Hygromycin, respectively^22^. The resulting cells were then transduced with lentiviral vector (Lenti-sgRNA-MS2-Zeocin; Addgene #61427) inserted with gRNA targeting promoter sequences of human BMP1 or a sequence that does not align to the human genome as a control, and subsequently zeocin-selected to generate the final cell lines. The target gRNA sequences were designed using the SAM website (http://sam.genome-engineering.org). We tested six different gRNAs in BxPC3 cells and selected two gRNAs that led to the best overexpression of BMP1. Between the two gRNAs, only gRNA 1 adequately overexpressed BMP1 in AsPC1 cells, hence gRNA 1 was used in AsPC1 cells. The gRNAs selected are as follows:

BMP1 gRNA 1 GGGCCGGGCCGCGCGCCAGC;

BMP1 gRNA 2 GGGCCGGGACAGTGCTCGGC;

Ctrl CATATTCCCCAAACTTCCTG.

### Expression vectors and cell transduction

Lenti-human BMP1 was part of the CCSB-Broad lentiviral expression collection and ordered from Horizon (ID: OHS6085-213578434, Horizon).

All PCOLCE and COL1A1 cDNA were cloned into the pHAGE-IRES-blast vector (a kind gift from David Benjamin). Lenti-PCOLCE constructs (WT and mut) were generated by inserting geneblocks (IDT) containing wild-type and mutated PCOLCE cDNA as well as C-terminal 6xHis into the pHAGE-IRES-blast vector. Lenti-COL1A1 WT construct was cloned by PCR from the human COL1A1 cDNA fragment in the pBlueScript-COL1A1 cDNA construct (MHS6278-202808048 from Horizon); lenti-COL1A1 mut construct was cloned by first mutating the human COL1A1 cDNA (with the conserved BMP1 cleavage site at D^1219^ replaced by R^1219^) in the pBlueScript-COL1A1 cDNA construct and then moving the mutated cDNA into the pHAGE-IRES-blast vector. All cDNA fragments were fully sequenced by Sanger sequencing prior to further use. As a control, pHAGE-IRES-blast vector was used.

BxPC3, AsPC1, and PANC1 cells were rendered zsgreen-positive by retroviral infection with MSCV-puromycin-IRES-Zsgreen modified from MSCV-IRES-Hygro as described previously^61^. Retroviral and lentiviral production and transduction of cells was performed as previously described^61^. WT and mut COL1A1 cell lines were selected single cell clones with comparable levels of COL1A1 cDNA expression.

### shRNA knockdown studies

miR30-based shRNAs targeting COL1A1 for knockdown were designed using a tool developed by the lab of Michael Hemann (shrna.mit.edu) and cloned into MSCV-Puro-miR30, as previously described^61^. shCOL1A1 was designed in the 5′UTR region of COL1A1. shFF is a control hairpin that recognizes sequence in the firefly luciferase gene (which does not exist in the cells). Cells expressing shCOL1A1 and shFF were selected with Puromycin. The target site for shCOL1A1 was TTGCATTCATCTCTCAAACTTA.

### Orthotopic implantation, subcutaneous implantation, tail-vein injection, and quantification of metastasis

For xenograft tumor experiments, 1 × 10^5^ AsPC1, BxPC3, or PANC1 cells in 50 μl PBS were injected into the pancreas of 8–10-week-old NOD/SCID/IL2Rγ-null (NSG) mice (Jackson Laboratory). In the PCOLCE experiment, 0.5 × 10^5^ PCOLCE-expressing cells and 0.5 × 10^5^ BMP1-expressing cells were mixed in 50 ul PBS for injection. Tumors were harvested 7 weeks (AsPC1 oe-BMP1, BxPC3 shBMP1, BxPC3 shCOL1A1/lenti-BMP1, BxPC3 PCOLCE set, BxPC3 COL1A1 WT/mut set), 8 weeks (BxPC3 oe-BMP1) or 9 weeks (PANC1 lenti-BMP1) post-injection.

For subcutaneous injection, 5 × 10^5^ in-vivo-lung-selected BxPC3 G1.1 cells^17^ in 50% Corning Matrigel (GFR, 356231, Corning) were injected into the flank of 8–10-week-old NSG mice. One week later, UK383367 was freshly dissolved in 15% SBE-β-CD (NC0557467, Fisher), filter sterilized, and injected peritumorally at 1 mg in 100 μl injection volume per mouse. The tumors were dosed every other day for a total of five times. Sham-treated mice were injected with 100 μl 15% SBE-β-CD. Mice were killed at the indicated end points.

Primary tumors were dissected, weighed and fixed overnight in 4% paraformaldehyde. Lungs and livers were collected and imaged with a Leica M165 FC dissecting microscope, and subsequently fixed overnight with 4% paraformaldehyde. Samples were kept in 70% ethanol prior to embedding and sectioning.

For tail-vein injection, 0.5 × 10^5^ cells in 100 μl PBS were injected into the lateral tail vein of 8- to 12-week-old NSG mice. The mice were killed 7 and 5 weeks later for the BxPC3 BMP1 oe set and shBMP1 set, respectively, and lungs were imaged with a Leica M165 FC dissecting microscope, and subsequently fixed overnight with 4% paraformaldehyde. Samples were kept in 70% ethanol prior to embedding and sectioning.

ZsGreen-positive metastatic load was quantified using ImageJ. Best thresholding method was chosen for each experiment and applied to all images for that experiment. Manual curation was applied when necessary. Two-tailed Student’s *t* test was performed to evaluate the statistical significance of the results. The normalized metastasis load was represented by the fraction of Zsgreen-positive area in the lungs or liver normalized to primary tumor weight.

### Cell growth assay

The assays were performed in the Incucyte Zoom System (Essen Bioscience). In all, 10,000 cells were seeded in triplicate into 96-well plates targeting for 10% confluency. In case of ascorbic acid treatment, cells were plated in corresponding culture medium with ascorbic acid of indicated concentration. Medium was carefully changed every 2 days during the growth assay. Phase-contrast images were captured every 3 h to calculate percent confluency. Doubling time was calculated using GraphPad prism non-linear regression curve fitting and was statistically tested by two-tailed Student’s t test.

### COL1A1 mutation genotyping

RNA was isolated from cells that expressed shCOL1A1 and WT or mut COL1A1 using the RNeasy kit (Qiagen) and cDNA was synthesized by reverse transcription using the First-Strand cDNA Synthesis Kit (Promega). PCR was performed using cDNA or COL1A1 WT/mut constructs (as controls) as a template with primers: Forward, AAAGATGGACTCAACGGTCTC; reverse, CTTCCAGTCAGAGTGGCACATCTTGAG. PCR products were purified with QIAquick kit (28104, Qiagen). Half of the PCR product remained undigested (uncut), and the other half was digested by NruI-HF (NEB) at 37 °C overnight (cut), then both were run on DNA gel side-by-side. The full-length PCR product is 356 bp, the mutation-containing sequence can be digested by NruI-HF into 253 bp and 103 bp while wild-type sequence cannot be digested. The percentage of mutated transcripts was estimated by the band intensity of the full-length 356 bp band in the “cut” lane divided by that in the “uncut” lane.

### Details of the following materials and methods are shown in Supplemental Information

proteomics, IHC quantification, cell immunofluorescence staining and quantification, reverse transcription and quantitative real-time PCR (primer info is in Supplementary Data 2), PCOLCE protein purification, procollagen processing assays, hydroxyproline GC-MS analysis, transmission electron microscopy.

### Reporting summary

Further information on research design is available in the Nature Research Reporting Summary linked to this article.

## Supplementary information

Supplementary Information

Description of Additional Supplementary Files

Supplementary Data 1

Supplementary Data 2

Reporting Summary

## Data Availability

The authors declare that all data supporting the findings of this study are available within the paper and the supplementary information. Proteomic data was published before^11^. The raw mass spectrometry data have been deposited in the public proteomics repository MassIVE (http://massive.ucsd.edu) using the identifier: MSV000082639. A reporting summary for this article is available as a Supplementary Information file.
