# Supplementary material for: Suppression of pancreatic ductal adenocarcinoma growth and metastasis by fibrillar collagens produced selectively by tumor cells
Source: Nat Commun. 2021 Apr 20;12:2328. doi: 10.1038/s41467-021-22490-9 (PMC8058088; doi:10.1038/s41467-021-22490-9)

Supplementary information

1. Supplementary Materials and Methods:

2. Supplementary figures 1-7

3. P values

4. Uncropped western blotting images

1. Supplementary Materials and Methods:

Proteomics:
Our proteomics experiments were previously published and described in detail[11]. Briefly, a TMT10 multiplexed isobaric labeling strategy was used to study a cohort of pancreatic tissues from human patients and a cohort of tissues from mice using the KPC genetic model system (LSL-KrasG12D/+;LSL-Trp53R172H/+;Pdx-1-Cre). Each cohort contained 18 tumors of varying types (normal, PanIN, Pancreatitis, PDAC) analyzed in two TMT10-plexes containing 9 individual samples and a common control (mix of the 18 samples) to bridge the plexes. ECM-enriched fractions were processed by solubilization in 8M urea, reduction and alkylatation of disulfide bonds, deglycosylation of proteins with PNGaseF, digestion with Lys-C and trypsin, and labeling with TMT10 reagents. After mixing the individual samples and common controls, the TMT10 plex peptide mixtures were fractionated by basic reversed phase and analyzed by LC-MS/MS with a Thermo Fisher QExactive Plus Orbitrap mass spectrometer. Identification and quantitation of the peptides and proteins in the MS/MS spectra utilized Spectrum Mill. The quantitation used log2 ratios of the TMT reporter intensity for each sample relative to the common control in each peptide spectrum match (PSM) with protein level TMT log2 ratios calculated as the median of all PSM level log2 ratios contributing to the protein. To account for any differences in ECM enrichment of the samples, the protein ratio distribution for each sample was normalized by the mean TMT ratios of the most abundant fibrillar collagen subunits, α1(I), α2(I) and α1(III).
For the domain analysis to identify ECM-protein isoforms (Figure 1 A-B, S1 A-B), we mapped the log2 ratios for each peptide identified by the TMT MS analyses (y axis) to their locations in the proteins (x axis) to detect regions that are over- or under-represented at different PDAC progression stages. For ease of illustration and to focus on domain representation rather than protein abundance changes, the log2 ratios of each peptide were normalized first to the overall protein abundance and further to that of the normal pancreas. The same normalization was applied to peptides in the analysis in Supplementary Figure 6A, then the log2 ratios were converted to fold changes. The normalized fold changes of the peptides from left and right side of the BMP1 cleavage site were plotted as mean+/-STDEV. Two-tailed Student's t test was performed to compare the differences.
For the intensity-based quantitation (Figure 1F and Supplementary Figure 1D) the peptide MS1 precursor ion intensity was demultiplexed in proportion to its individual peptide-level TMT reporter ion ratio. The experiments involving the xenograft cell lines (AsPC3, BxPC3, HuO1, and HuO2) were performed as label-free analyses (Figures S1E-F), so the peptide MS1 intensity-based quantitation did not require demultiplexing. For estimation of the percentage of pro-domain to mature protein in the ECM (Figures 1D and S1F), the percentage was calculated by the average intensity of peptides that map to the prodomain region divided by that of the mature collagen region. While the MS ionization responses of equimolar amounts of different peptides are variable, the average intensities of multiple peptides from different regions provide a metric with a consistent trend between sample types and an estimate of its magnitude. The same considerations apply for the species-specific peptide intensity-based metric (Supplementary Figure 1E). All peptides analyzed in these analyses are peptides unique

to a given protein. Peptides that are mapped to more than one protein were removed from our analyses.

Immunohistochemistry:
Briefly, tissues (lungs and pancreas) were fixed in 4% paraformaldehyde or 10% neutral-buffered formalin at room temperature (RT) overnight and paraffin-embedded following standard procedures. Consecutive sections were prepared using a Leica RM2255 rotary microtome (Leica Biosystems) and dried at 60°C for 1h. The sections were then dewaxed and rehydrated before staining with hematoxylin and eosin (H&E) or treatment with heat-induced epitope-retrieval using decloaking chamber (Biocare medical) prior to immunostaining. The sections were incubated in 10 mM sodium-citrate (pH6.0) or 10mM Tris (pH9.0) buffered solutions containing 0.05% Tween at 120°C for 2min using a pressure cooker. If needed, additional enzyme treatment using pepsin was included. To obtain consistent and reliable staining on all tissues investigated, an automated staining system (LabVision Autostainer 360, ThermoFisher) was used. To efficiently destroy all endogenous peroxidase and alkaline phosphatase activity in the tissue, the sections were subsequently pretreated using BLOXALL endogenous enzyme blocking solution (Vector Laboratories) for 10 min. After a blocking step with normal serum, the sections were incubated with the individual primary antibodies for 1h followed by secondary ImmPRESS polymer detection systems (Vector Laboratories) according to the manufacturer's protocol. The Vulcan Fast Red Chromogen Kit 2 (red staining; Biocare Medical) and the DAB Quanto Substrate System (brown staining; ThermoFisher) were applied as substrates. For multi-color IHC stainings, following HIER and blocking steps, the individual antibodies were incubated consecutively using the chromogens indicated above. For counterstaining hematoxylin was used.

Quantification of IHC
To assure consistent and comparable IHC results, tumors from each experiment were assembled onto a tissue microarray and stained together. For one color quantification, the scanned IHC screenshots (20x) were processed through Fiji. Briefly, images were first color-deconvoluted using program "FastRed FastBlue DAB" to separate the red color channel which shows antibody staining. Collagen staining was measured with "Mean gray value" in the red color channel. The intensity score was calculated by log(255/Mean gray value) for the 8-bit images. Three representative fields were measured per tumor and averaged to give a score for each tumor. For the C-pro extracellular signal fraction quantification, the scanned IHC images were also processed through Fiji. Briefly, the mesenchymal region was first selected in each image, images were then color-deconvoluted using program "FastRed FastBlue DAB", and then the red color channel that represents C-pro staining and the brown channel representing VIM staining were thresholded using "Default" and changed to binary mode. The binary pictures for the two channels were then added together under "image calculator". The positive area fractions in VIM channel (a) and the combined channel (b) were measured and then calculated by the equation (b-a)/a and lastly normalized to that of normal pancreas. Three representative fields were measured per tumor and averaged to give a score for each tumor. The tumor intensity scores were plotted for each condition and statistically tested by two-tailed Student's t test.

Ki67 staining was quantified by first thresholding the red channel by "default", then applying "Fill holes" and "Watershed", and finally using "Analyze particles" to count the number of positive cells per unit area. Three representative fields were measured per tumor and averaged to give a score for each tumor. Cleaved caspase 3 positive cells are sparse, so the numbers of positive cells per image were blinded and manually counted. Ten representative fields were scored per tumor and averaged to give a score for each tumor.

Cell immunofluorescence staining and quantification

Coverslips were coated with 20ug/ml FN1 (4305-FNB, R&D systems) in PBS for 1 hour at RT. hT1 and hM1 cells were then directly cultured on the FN1-coated coverslips housed in 12-well tissue culture plates in DMEM media with 10% FBS till confluency. Upon confluency, cell media were replaced with DMEM+10%FBS+0.1mM ascorbic acid with 0μM, 5μM or 20μM UK383367 (S2224, SelleckChem), or 1μg/ml BMP1 (1927-ZN-010, R&D systems). Cell media were replaced with fresh media every other day for a total of 8 days.

Cells grown on the coverslips were fixed with freshly prepared 4% paraformaldehyde in PBS for 5min at RT and stained with primary and secondary antibodies, washed with PBS in between, and counterstained with DAPI in PBS with 0.1% Triton X-100. The primary antibodies used were human ColI/α1(I) (AF6220, R&D systems, 1:100); C-pro/α1(I) (600-401-D19, Rockland, 1:200). Images were taken with a Nikon A1R laser-scanning confocal microscope using a 63x objective. Images were quantified in ImageJ. Thresholding method was chosen and applied to represent fluorescence signals in all images. For a given image, the C-pro/α1(I)-positive area was divided by the α1(I)-positive area to give an area fraction number that represents C-pro/α1(I) signal on α1(I) fibrils. At least 17 images were quantified for each condition. Two-tailed Student's t test was performed to evaluate the statistical significance of the results.

Reverse transcription and quantitative real-time PCR

RNA was isolated from cell or tumor lysates using the RNeasy kit (Qiagen) and cDNA was synthesized by reverse transcription using the First-Strand cDNA Synthesis Kit (Promega). qPCR reactions were performed using Bio-Rad SYBR Green Supermix (Bio-Rad) according to the manufacturer's instructions. Student's t test was performed to evaluate the statistical significance of differences between groups. Human and murine PCR and qPCR primers used are listed in Supplemental table 2.

PCOLCE protein purification

293FT cells stably expressing lenti-PCOLCE (WT with C-terminal 6-His) and lenti-PCOLCE (mut with C-terminal 6-His) constructs were generated. Supernatants from the cultured 293FT cells were collected, cleared of cell debris, concentrated and buffer exchanged in Amicon Ultra-15 (Millepore), and then passed through columns packed with Ni-NTA resin (30210, Qiagen, Germantown, MD) for protein purification according to the native condition purification method in the ProBond purification system manual (Invitrogen, Carlsbad, CA). The resulting purified proteins were run on 4-20% SDS-PAGE and silver stained (24612, ThermoFisher). The protein concentration was measured by Pierce BCA kit (23227, ThermoFisher).

Procollagen processing assays

In the time-course cleavage experiment, 10ng/μl rhmini-proα1(I) (6220-CL-020, R&D systems) was digested in assay buffer (50mM Tris-HCl, pH 7.5, 5mM CaCl2, 150mM NaCl) with 1ng/μl BMP1 (1927-ZN-010, R&D systems) and 0.7ng/μl WT or mut PCOLCE. Reactions were incubated at 37°C for 30min. Aliquots were removed from each reaction at the indicated time points, and the reaction was stopped by addition of SDS, dithiothreitol (DTT), and EDTA to final concentrations of 2% SDS, 0.1M DTT, and 10mM EDTA.

Hydroxyproline GC-MS analysis

About 2mm^3 of xenograft tumor samples were snap frozen. Necrotic tumor cores and tumor capsule regions were carefully avoided. Acid protein hydrolysis of tissue was performed as described previously [62]. Frozen tissue was boiled for 24 hours at 100°C in 1mL of 6M HCl for amino acid analysis (84429, Sigma). 50µL of HCl solution was then dried under nitrogen gas while heating at 80°C. Dried hydrolysates were stored at -80°C until derivatization.
Metabolites were analyzed as described previously [63]. Dried protein hydrolysates were re-dissolved in 16µL HPLC grade pyridine (Sigma 270407) prior to derivatization with 20µL N–methyl–N–(tert–butyldimethylsilyl)trifluoroacetamide + 1% tert–butyldimethylchlorosilane (375934, Sigma) at 60°C for 1 hour. Following derivatization, samples were analyzed using a DB-35MS column (Agilent Technologies) in an Agilent 7890 gas chromatograph coupled to an Agilent 5975C mass spectrometer. Helium was used as the carrier gas at a flow rate of 1.2 mL/min. One microliter of sample was injected at 270°C. After injection, the GC oven was held at 100°C for 1 min. and increased to 300°C at 3.5 °C/min. The oven was then ramped to 320°C at 20 °C/min. and held for 5 min. at this 320°C. The MS system operated under electron impact ionization at 70 eV and the MS source and quadrupole were held at 230°C and 150°C, respectively. The detector was used in scanning mode, and the scanned ion range was 100–650 m/z.

Transmission electron microscopy

Xenograft tumor pieces were fixed with 2% glutaraldehyde, 2.5% paraformaldehyde in 100mM cacodylate buffer for 1 hour at 4°C, post-fixed in 2% osmium tetroxide in Veronal acetate buffer for 1 hour at 4°C. They were then en-bloc stained with 2% uranyl acetate in sodium maleate buffer overnight in the dark, and serially dehydrated with ethanol, then embedded in resin at 60°C for 48 hours.
Embedded samples were trimmed by hand and then sectioned on a Leica UC7 ultramicrotome. Semi-thin sections of 0.5 to 1.0 micrometers thickness were mounted on glass slides and used to evaluate sample morphology and orientation. Ultrathin sections of 60nm thickness were floated onto distilled water and collected on copper grids with a carbon-coated nitrocellulose support film. The sections were stained with 2% uranyl acetate and lead citrate before imaging in the microscope.
The TEM grid with sectioned specimens was mounted on a JEOL single-tilt holder equipped in the TEM column. The grid was cooled down by liquid nitrogen and imaged on a JEOL 2100 FEG microscope using minimum dose method that was essential to avoid sample damage under the

electron beam. The microscope was operated at 200 kV and with magnification in the range of 10,000~60,000. All images were recorded on a Gatan 2kx2k UltraScan CCD camera.

Collagen fibril diameter analysis was obtained from cross-sectioned collagen fibrils on nonoverlapping images taken from two independent tumors in each group and then pooled by genotype, with at least three images taken and 60 fibrils measured per tumor. All fibrils on a given image were measured. Images were masked before fibril diameters measurement using ImageJ. Fibril diameters were measured along the minor axis of the fibril cross section.

2. Supplementary figures:

Supplementary Figure 1. Mass spectrometry (MS) of enriched ECM from progressive PDAC stages reveals retention of fibrillar collagen prodomains

A,B, peptide analysis showed higher abundance of peptides located in [1] the N-prodomain of proα1(V) (in which N-pro domain, rather than C-prodomain is a target of prodomain protease) and [2] the C-prodomain of proα2(V) in both human and mouse PDAC ECM as compared with normal pancreas ECM. Peptides are plotted with their fold change relative to normal (y axis) and starting amino acid location (x axis). Protein isoform domain structures from SMART (smart.embl-heidelberg.de) are displayed at the bottom. Green shade indicates N-prodomains and orange shade indicates C-prodomains. ****, $p<0.0001$. P-values come from two-tailed t-tests between the log2(fold change) in peptides from the prodomain regions and from the mature collagen region.

C, double color immunohistochemical staining showing that α-C-pro/α1(I) (LF42) staining is present in the extracellular space (negative for the cytoplasm marker VIM) in PDAC but not in normal pancreas in human. Arrows point to extracellular C-pro/α1(I) regions. The images are representative of 3, 3, 3, 7 tissue samples (left to right).

D, intensity of peptides that map to human C-pro/α1(I) (N=7). Red marks represent the peptide that spans the pC-protease cleavage site (sequence shown) in human proα1(I).

E, using xenograft tumor ECM MS peptide data to estimate percentage of fibrillar collagens that are deposited by cancer cells (human sequence) or stromal cells (mouse sequence) in three types of collagens. The percentage was calculated by the total intensity of the human sequence peptides by the total intensity of all peptides using xenograft tumor ECM MS peptide data.

F, using xenograft tumor ECM MS peptide data to estimate the percentage of retained C-prodomain in collagen III from cancer cells vs. stromal cells in the ECM of xenograft tumors. The percentage was calculated by the average intensity of the species-specific peptides that map to the prodomain region divided by that of the mature collagen region. All columns are represented by mean ± SD.

Supplementary Figure 7

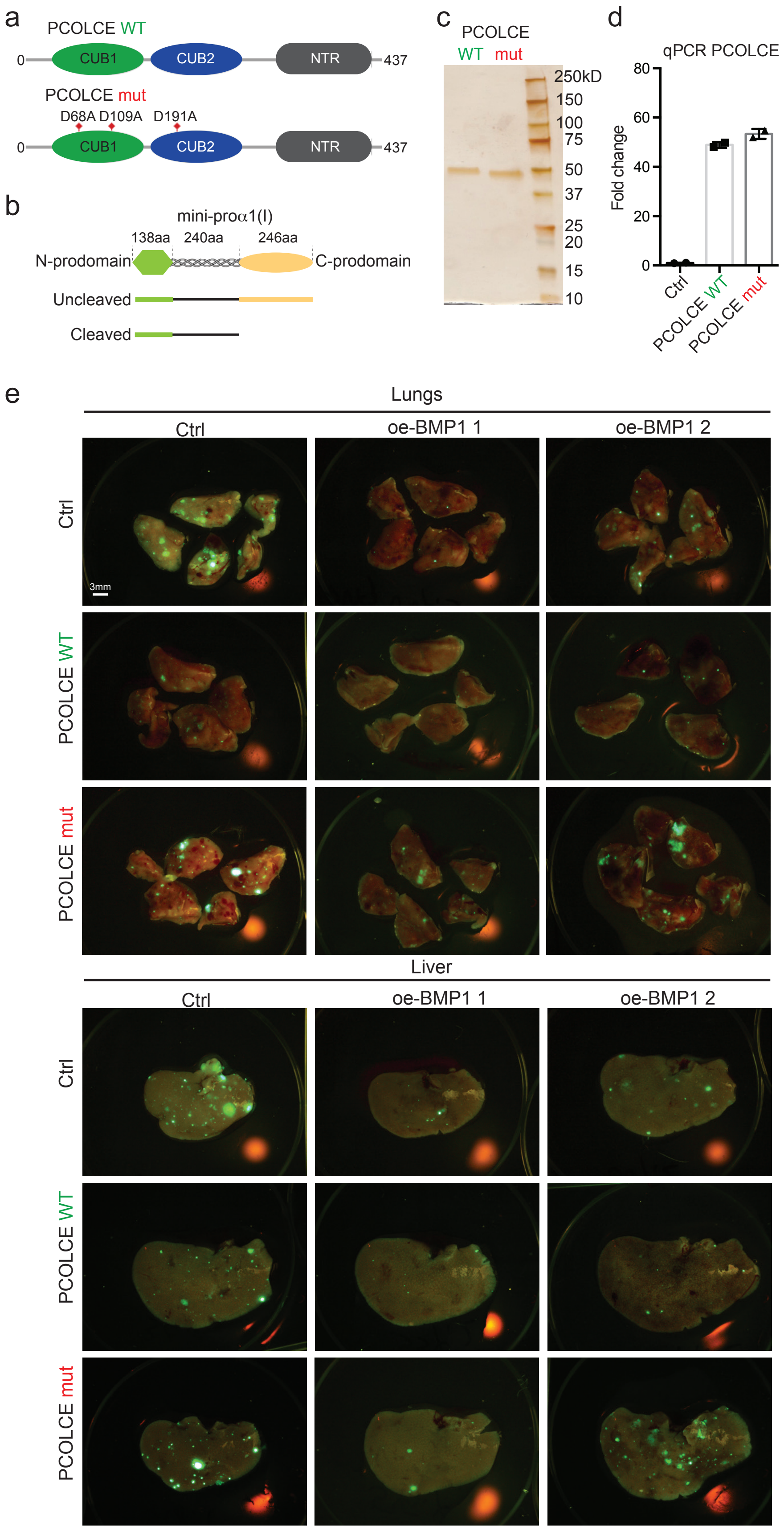

Supplementary Figure 2. BMP1 splicing isoforms and testing of BMP1 inhibitor, UK383367, in fibroblast cultures *in vitro*.

A, BMP1 short (BMP1-1) and long (BMP1-3, also named mTolloid) splicing isoform schemata.

B, CRISPR-activation induced the expression of both BMP1 isoforms, using qPCR primers that specifically recognize the short and long forms of BMP1 (N=2). In all other BMP1 qPCR experiments, BMP1 primers that recognize both isoforms were used.

C, D, tail-vein injection of BxPC3 cells oe-BMP1 (C) or shBMP1 (D) led to reduced and increased pulmonary metastasis, respectively. N=5 for all columns.

E, F, increasing amounts of BMP1 inhibitor UK383367 increased extracellular C-prodomain (C-pro/α1(I)) signal (E) after staining without permeabilization, quantified by the area fraction of mature α1(I) that is also positive for C-pro/α1(I) (F). On the contrary, adding BMP1 protein decreased C-pro/α1(I) signal (E,F). N numbers are 18, 17, 20, 19 for hT1 set and 20, 20, 15, 17 for hM1 set.

G, H, I, increasing amounts of BMP1 inhibitor UK383367 increased pCα1(I) to mature α1(I) ratio and decreased mature α1(I) expression, whereas adding BMP1 protein had the opposite results in both hT1 (G) and hM1 (H) cells. Quantification is shown in I (N=3).

J, cultured lung-selected BxPC3G cells deposit less Col I compared with control BxPC3 cells when treated with UK383367. J shows images representative of three independent experiments with β-actin as a sample processing control. The quantified WB signal is represented as mean ± SD.

K, Ki67 and cleaved caspase 3 (CC3) staining in xenograft tumors. The quantification of IHC signal was shown on the right hand side. N numbers are 4 and 5 from left to right for both Ki67 and CC3 staining quantifications.

All p-values come from two-tailed Student's t-tests. All columns are represented by mean ± SD.

Supplementary Figure 6

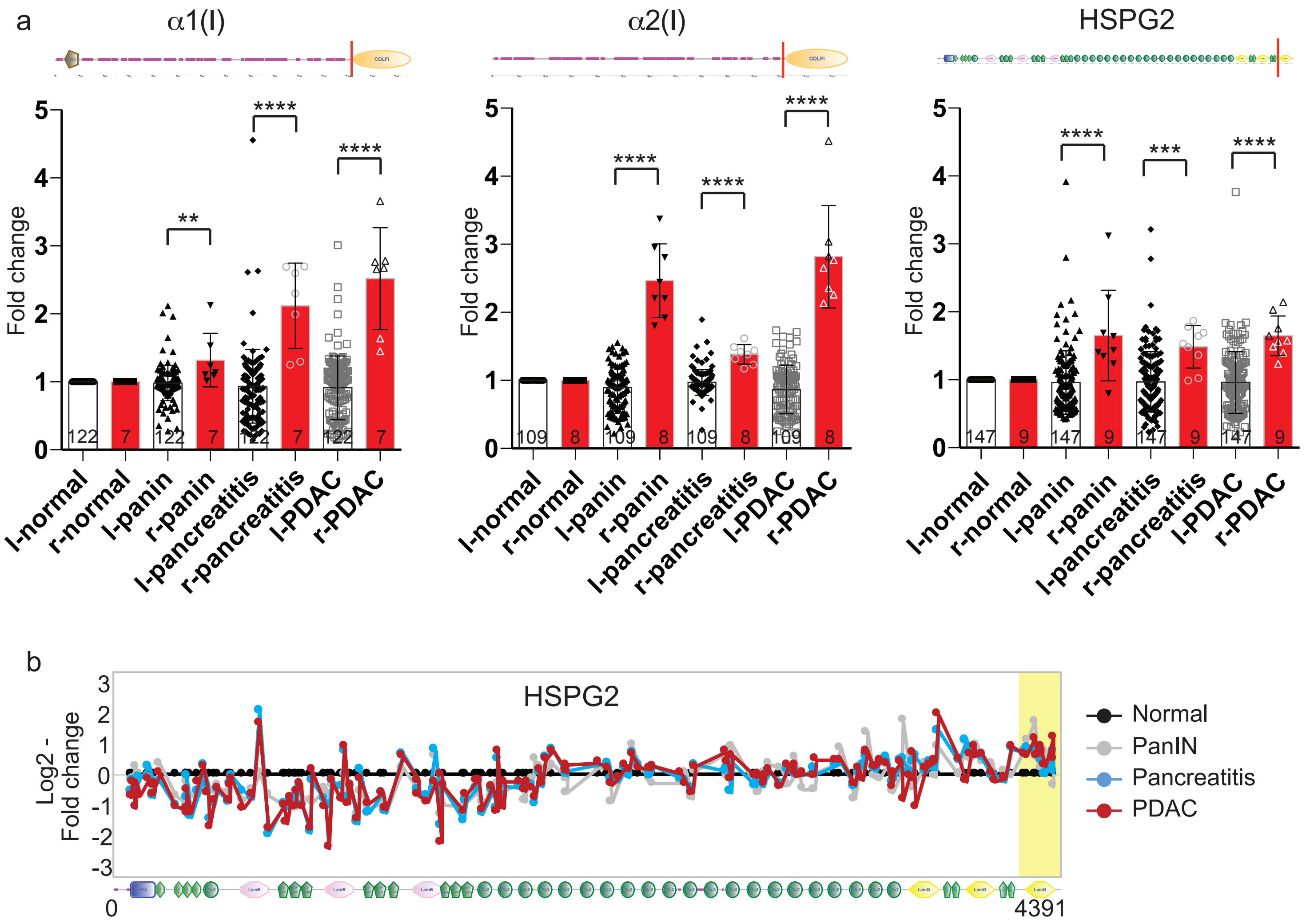

Supplementary Figure 3. Test of species-specific Collagen 1 antibodies and measurement of collagen fibrils in xenograft tumors.

A, western blotting of enriched human and mouse ECM indicated species-specificity of the ColI antibodies. Representative images were shown from two independent repeats.

B, IHC on human and mouse kidneys and intestines showed species specificity of the ColI antibodies. Images are representative of two independent staining experiments.

C-E, transmission electron microscopic analysis of collagen fibril width in xenograft tumor ECM showed reduced collagen fibril widths in BMP1-overexpressing tumors. Representative cross-sectional and longitudinal-sectional images are shown in C. The fibril width distribution and average diameter were shown in D and E, respectively. N numbers are 207, 191, 211 from left to right (see also Supplemental methods). All p-values come from two-tailed Student's t-tests. All columns are represented by mean ± SD.

Supplementary Figure 5

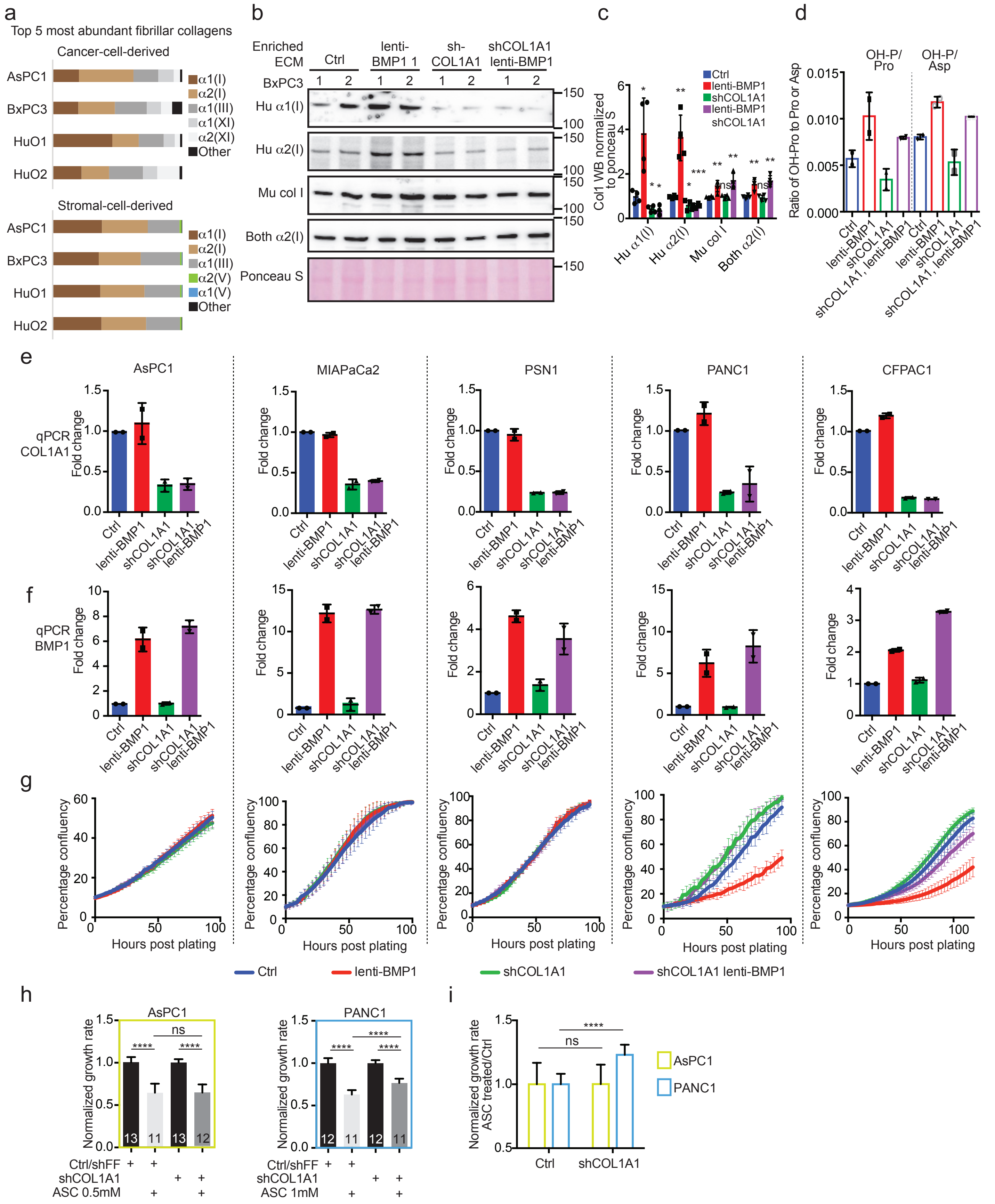

Supplementary Figure 4. BMP1 overexpression in AsPC1 xenograft tumors

A-E, orthotopic injection of AsPC1 cells (A) overexpressing BMP1 using CRISPR-activation method (B) resulted in unaltered primary tumor weight (C), and lung metastasis load (D). Lung metastasis images are shown in E. Note that AsPC1 cells are not metastatic to the liver. Mouse numbers are 6 and 5 (left to right).

F, G, cultured oe-BMP1 AsPC1 cells express (low) levels of α1(I) protein (F) and mRNA (G) comparable to control. F shows images representative of three independent experiments with β-actin as a sample processing control. The quantified WB signal is represented as mean ± SD.

H, I, IHC on xenograft tumor sections showed no change in ColI levels in the AsPC1 oe-BMP1 cells. The intensities were quantified in I (N numbers are 6 and 5, left to right).

J, K, WB on enriched ECM samples from xenograft AsPC1 tumors showed no change in either cancer-cell-derived (human) or stroma-derived (mouse) ColI deposition upon BMP1 overexpression. K is the quantification of the band intensities of four tumors (two were shown in J and two came from an independent repeat).

L, the normalized intensity of protein Hypro is not changed in oe-BMP1 AsPC1 tumors (N numbers are 4 and 3, left to right).

M-Q, BMP1 is overexpressed in the oe-BMP1 xenograft tumors compared to control tumors by IHC (M, N=6 and 5, left to right) and species-specific BMP1 qPCR (Q) using human-specific BMP1 and β-actin primers that are validated in panels N-P (N=2 for N-Q). All p-values come from two-tailed Student's t-tests. All columns are represented by mean ± SD.

Supplementary Figure 4

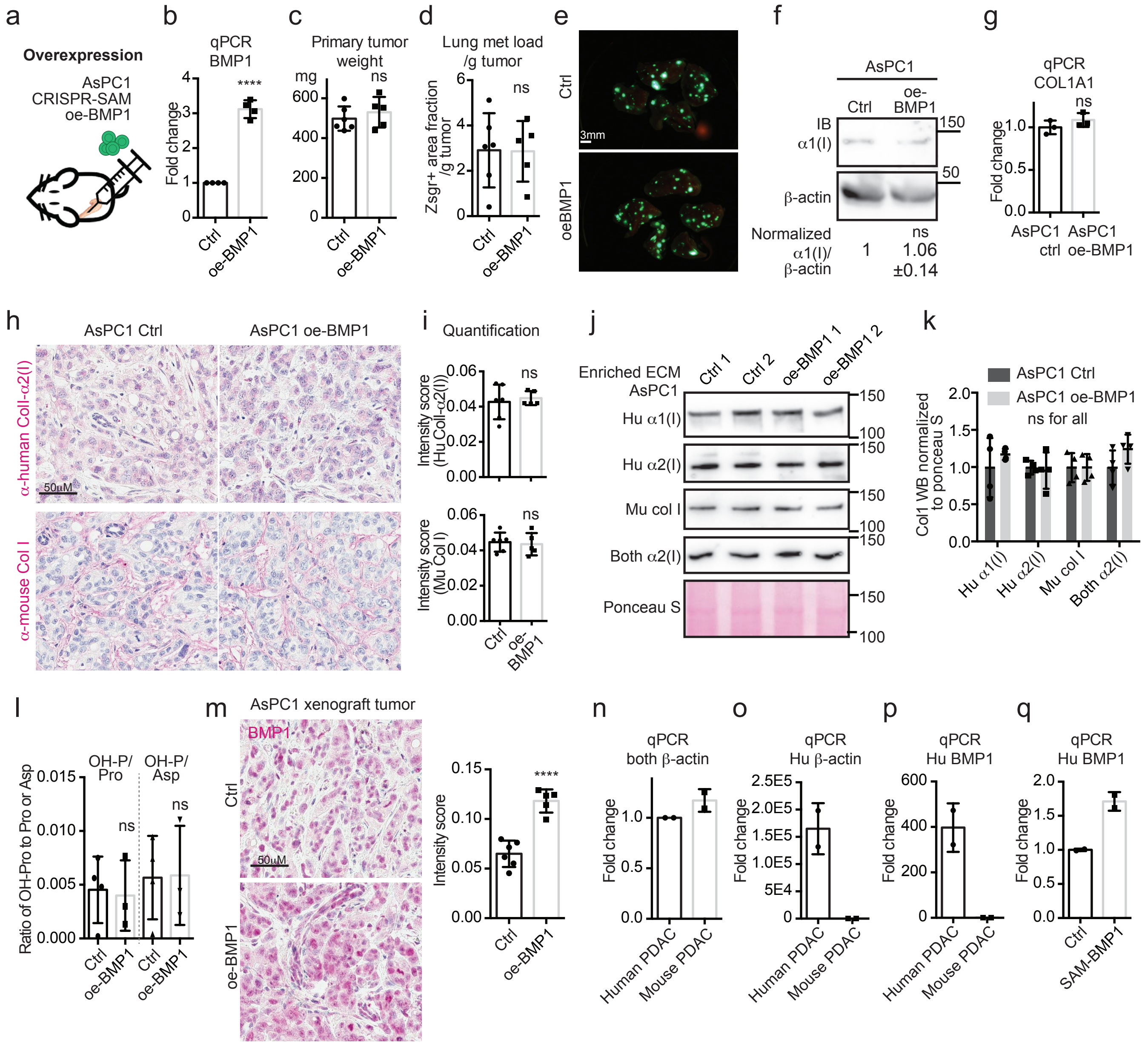

Supplementary Figure 5. Extended collagen quantification in lenti-BMP1 and/or shCOL1A1 tumors.

A, analysis of protein abundance for all the fibrillar collagens in the xenograft tumor ECM showed that ColI is the most abundant fibrillar collagen species regardless of cell of origin. These xenograft tumor MS data were published previously[11].

B, C, WB of enriched ECM samples from xenograft BxPC3 tumors showed that cancer-cell-derived ColI was increased by lenti-BMP1 but reduced by shCOL1A1, while stroma-derived ColI was increased by lenti-BMP1 and not changed by shCOL1A1. The intensities were quantified in C (N=4), color coding here and in panels C-G as in Fig. 5A.

D, the normalized intensity of protein Hypro to protein Asp is increased in lenti-BMP1 tumors (N=2).

E-G, generating AsPC1, MIAPaCa2, PSN1, PANC1, and CFPAC1 cells that either overexpress BMP1 or are knocked down for COL1A1 or both, as indicated by qPCR on COL1A1 (E, N=2) and BMP1 (F, N=2), and testing them for their growth, as indicated by the growth curves (G, refer to Figure 5L for N numbers).

H,I, ascorbic acid (ASC) suppressed *in vitro* cell growth in AsPC1 and PANC1 cells. Although different cell lines have different sensitivity to ASC concentration, using appropriate sensitive ASC concentration for each cell lines, the ASC suppression effect could be partially alleviated by knocking down COL1A1 expression in high endogenous COL1A1 expressing PANC1 cells, but not in low COL1A1 expressing AsPC1 cells, as quantified in I (N numbers are 11, 11, 12, 11, from left to right).

All p-values come from two-tailed Student’s t-tests. All columns are represented by mean ± SD.

Supplementary Figure 3

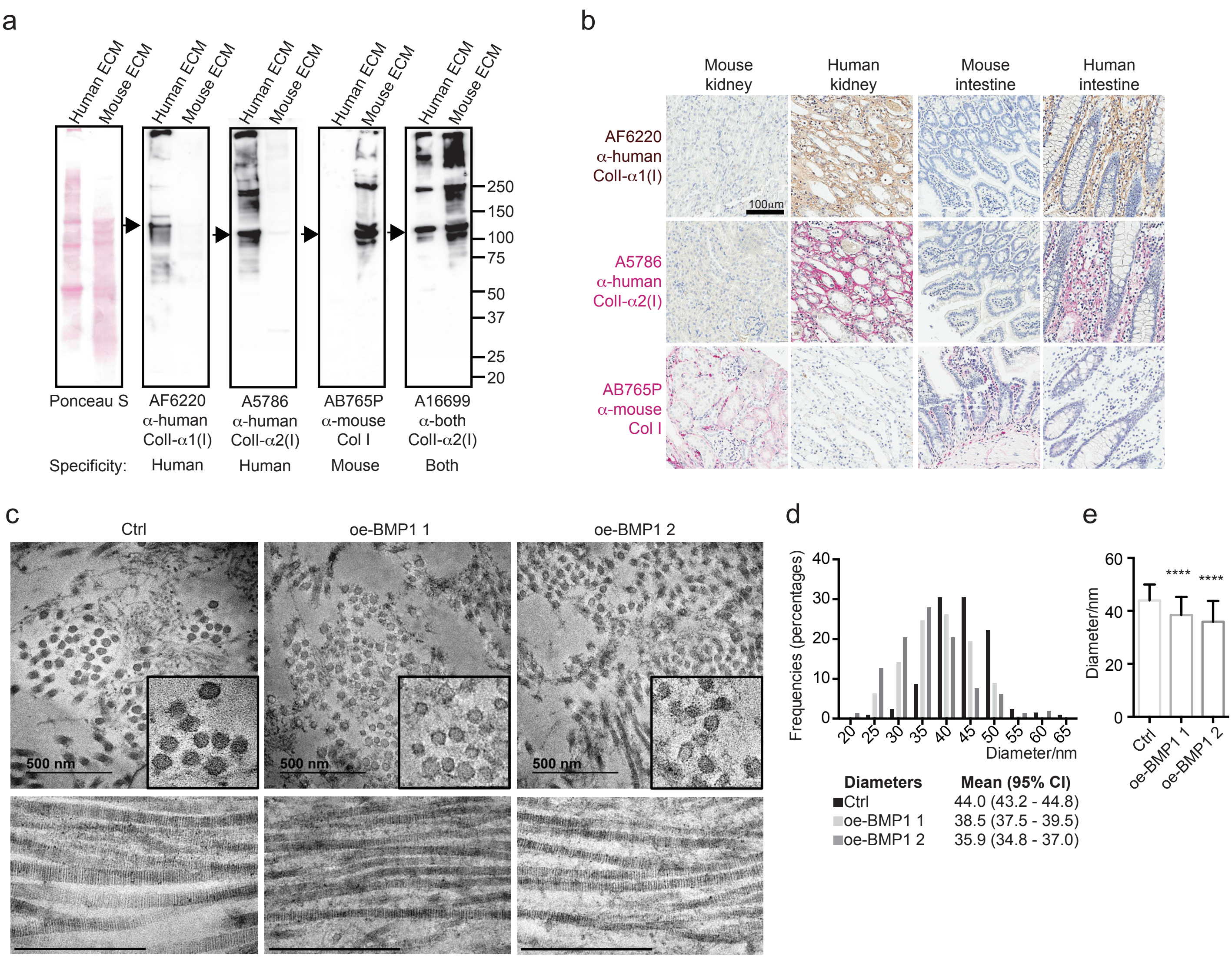

Supplementary Figure 6. Peptide analysis of other BMP1 targets.

A. The abundance of peptides located left (l-, white bar) and right (r-, red bar) of the BMP1 cleavage site, represented by normalized fold change compared to normal pancreas. Protein domain structures from SMART (smart.embl-heidelberg.de) are displayed at the top with the BMP1 cleavage site shown by the red bar. All p-values come from two-tailed Student's t-tests. All columns are represented by mean ± SD. Note that HSPG2 shows a real but smaller retention of the C-terminal domain as compared with procollagens. N numbers are indicated on the graphs.

B. Peptides from HSPG2 are plotted with their log2-fold change relative to normal (y axis) and starting amino acid location (x axis). Protein isoform domain structures from SMART (smart.embl-heidelberg.de) are displayed at the bottom. Yellow region indicates LG3 domain showing multiple peptides with elevated levels in this region.

Supplementary Figure 2

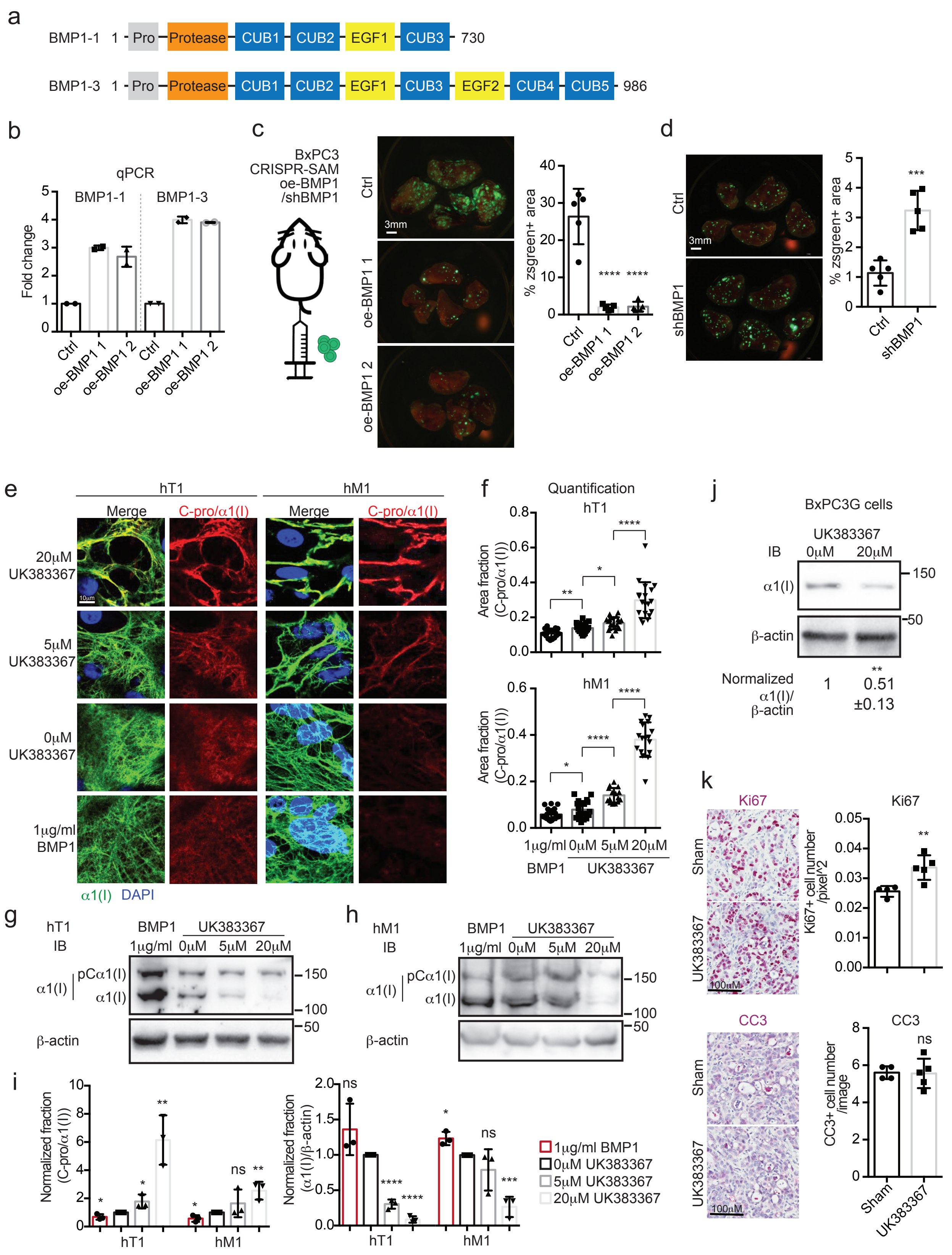

Supplementary Figure 7. Extended figure 6.

A, schematics for WT and mut PCOLCE with their protein domains (CUB1, CUB2 and NTR) and the identities and locations of three mutated residues shown.

B, schematics of the processing of mini-procollagen1A1, with the uncleaved (mini-proα1(I)) and cleaved forms shown.

C, silver staining of SDS-page loaded with purified WT and mut PCOLCE. Image is representative of three independent experiments.

D, qPCR of PCOLCE indicating comparable expression levels of WT and mut PCOLCE (N=2). Columns are represented by mean ± SD.

E, representative images showing the lung and liver metastasis from the orthotopic injected mice in Figure 6.

Supplementary Figure 1

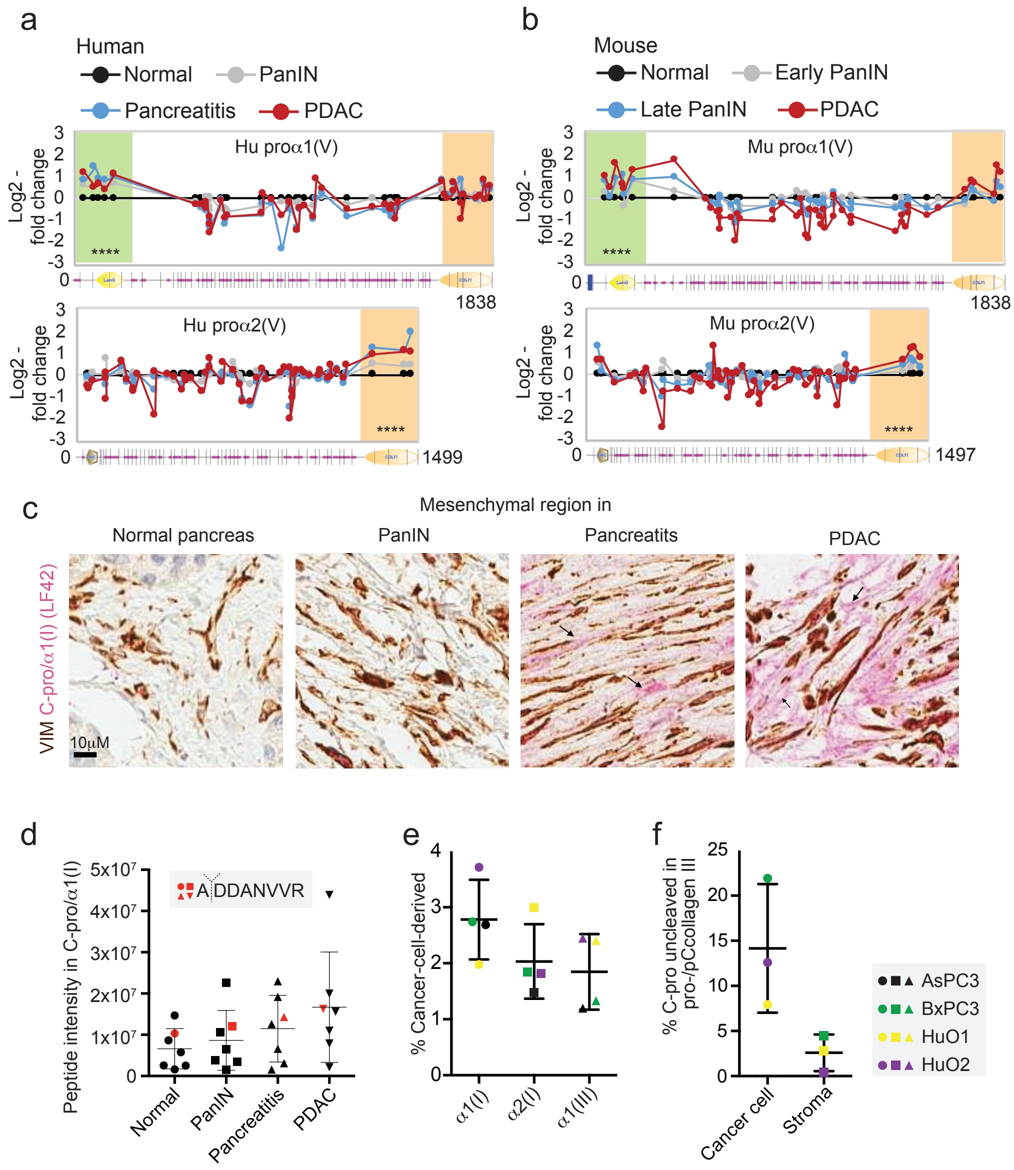

3. P values:

All p values are presented left to right (l to r) in each panel unless otherwise stated.

Figure 1	
D	p=0.0188
	p=0.0099

Figure 2	
E	p=0.0006
	p=0.0181
F	p=0.0005
	p=0.0025
G	p=0.0091
	p=0.0288
K	p=0.0076
L	p=0.0466
M	p=0.0098
P	p=0.0224
R	p=0.0432

Figure 3	
D top	p=0.0043
	p=0.0216
D bottom	p=0.0070
	p=0.0120
F	p=0.0024
	p=0.0278
	p=0.0232
	p=0.0370
	p=0.0452
K	p=0.0462
M	p=0.0028
	p=0.0007

Figure 4	
I	p=0.0002
K	p=0.0025
	p=0.0009
L	p=0.0302
	p=0.0105

Figure 5	
D	p=0.0206
	p=0.0352
F	p=0.0048
	p=0.0001
	p=0.0028
G	p=0.0012
	p=0.0417
	p=0.0050
H	p=0.0471
	p=0.0065
	p=0.0068
K top	p=0.0451
	p=0.0273
	p=0.0222
K middle	p=0.0245
	p=0.0063
	p=0.0304
K bottom	p=0.0222
	p=0.0427
L	p=0.0328
	p=0.0216
	p=0.0336
	p=0.0008

Figure 6		
C	column 4 and 5	p=0.0386
D	column 1 and 2	p=0.0062
	column 2 and 3	p=0.0333
	column 4 and 5	p=0.0381
	column 5 and 6	p=0.0456
	column 8 and 9	p=0.0028
	column 1 and 4	p=0.0020
E	column 1 and 2	p=0.0298
	column 4 and 5	p=0.0339
	column 5 and 6	p=0.0459
	column 7 and 8	p=0.0369
	column 8 and 9	p=0.0043
	column 1 and 4	p=0.0030
G	column 1 and 2	p=0.0456
	column 4 and 5	p=0.0036
	column 5 and 6	p=0.0003
	column 7 and 8	p=0.0028
	column 8 and 9	p=0.0001
	column 1 and 7	p=0.0048

Figure 7	
D	p=0.0022
F	p=0.0134
G	p=0.0457
K top	p=0.0009
K middle	p=0.0012
M (l to r)	p=0.0012
	p=0.0089

Figure S2	
B	p=0.0009
	p=0.0222
	p=0.0008
D	p=0.0003
F top	p=0.0042
	p=0.0123
F bottom	p=0.0391
I	p=0.0493
	p=0.0234
	p=0.0059
	p=0.0328
	p=0.0072
	p=0.0124
	p=0.0009
K	p=0.0090

Figure S5	
C (l to r)	p=0.0139
	p=0.0137
	p=0.0115
	p=0.0021
	p=0.0161
	p=0.0004

Figure S6		
A	p=0.0014	
	p=0.0008	

Figure S7	
D	p=0.0003
	p=0.0007

4. Uncropped western blotting images

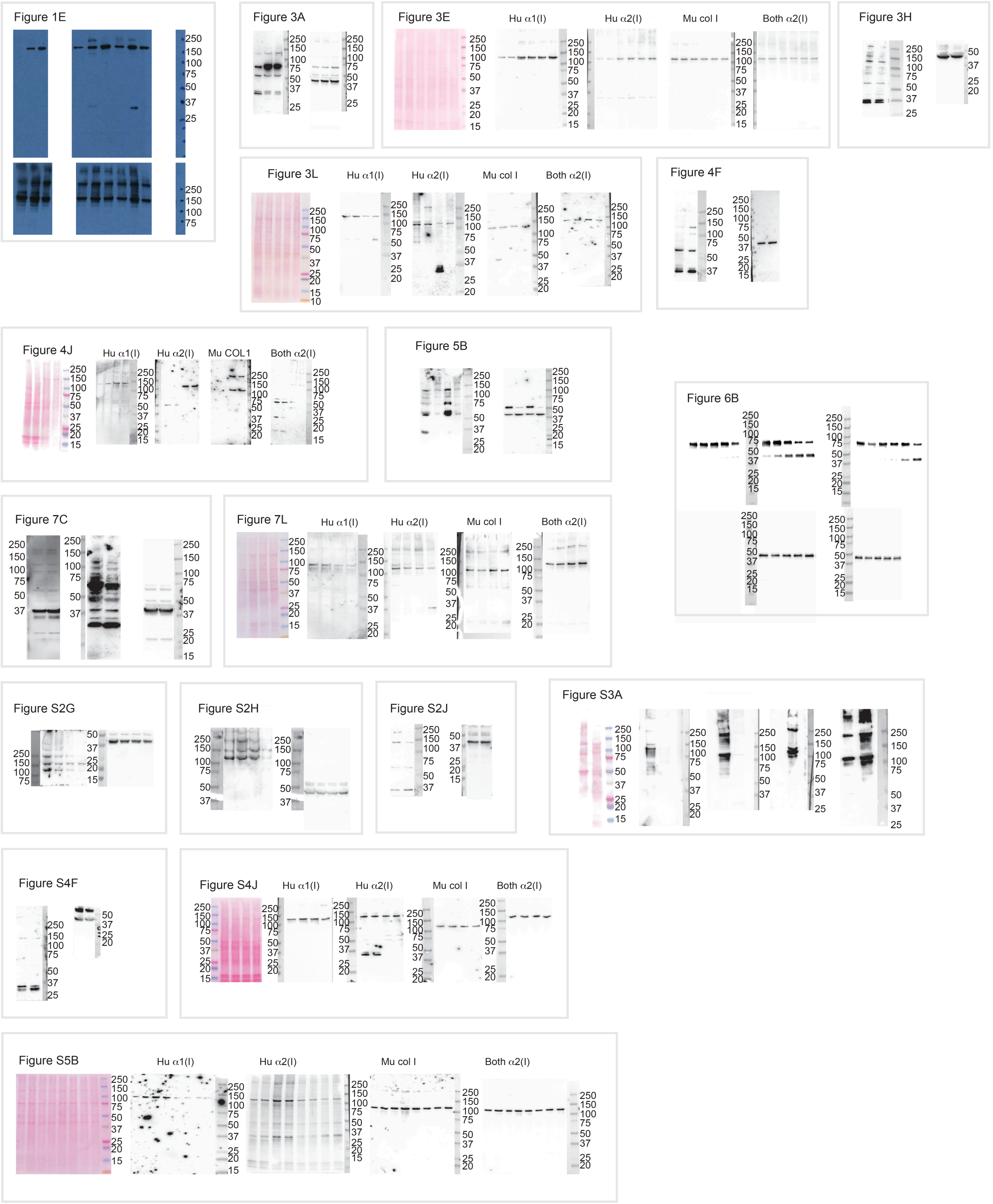

Supplement: Supplementary file 1 — Supplementary Information [file 41467_2021_22490_MOESM1_ESM.pdf]
