# Supplementary material for: Suppression of pancreatic ductal adenocarcinoma growth and metastasis by fibrillar collagens produced selectively by tumor cells
Source: Nat Commun. 2021 Apr 20;12:2328. doi: 10.1038/s41467-021-22490-9 (PMC8058088; doi:10.1038/s41467-021-22490-9)
Supplement: Supplementary file 2 — Description of Additional Supplementary Files [file 41467_2021_22490_MOESM2_ESM.docx]

File Name: Supplementary Data 1
Description: BMP1, TLL1, TLL2 coexpressed genes

File Name: Supplementary Data 2
Description: PCR and qPCR primers
