# Supplementary material for: Suppression of pancreatic ductal adenocarcinoma growth and metastasis by fibrillar collagens produced selectively by tumor cells
Source: Nat Commun. 2021 Apr 20;12:2328. doi: 10.1038/s41467-021-22490-9 (PMC8058088; doi:10.1038/s41467-021-22490-9)
Supplement: Supplementary file 5 — Reporting Summary [file 41467_2021_22490_MOESM5_ESM.pdf]

nature research

Corresponding author(s): Richard O. Hynes

Last updated by author(s): Jan 31, 2021

Reporting Summary

Nature Research wishes to improve the reproducibility of the work that we publish. This form provides structure for consistency and transparency in reporting. For further information on Nature Research policies, see our Editorial Policies and the Editorial Policy Checklist.

Statistics

For all statistical analyses, confirm that the following items are present in the figure legend, table legend, main text, or Methods section.

n/a	Confirmed	
☐	☒	The exact sample size (n) for each experimental group/condition, given as a discrete number and unit of measurement
☒	☐	A statement on whether measurements were taken from distinct samples or whether the same sample was measured repeatedly
☐	☒	The statistical test(s) used AND whether they are one- or two-sided<br>*Only common tests should be described solely by name; describe more complex techniques in the Methods section.*
☒	☐	A description of all covariates tested
☒	☐	A description of any assumptions or corrections, such as tests of normality and adjustment for multiple comparisons
☐	☒	A full description of the statistical parameters including central tendency (e.g. means) or other basic estimates (e.g. regression coefficient) AND variation (e.g. standard deviation) or associated estimates of uncertainty (e.g. confidence intervals)
☐	☒	For null hypothesis testing, the test statistic (e.g. F, t, r) with confidence intervals, effect sizes, degrees of freedom and P value noted<br>*Give P values as exact values whenever suitable.*
☒	☐	For Bayesian analysis, information on the choice of priors and Markov chain Monte Carlo settings
☒	☐	For hierarchical and complex designs, identification of the appropriate level for tests and full reporting of outcomes
☐	☒	Estimates of effect sizes (e.g. Cohen's d, Pearson's r), indicating how they were calculated

Our web collection on statistics for biologists contains articles on many of the points above.

Software and code

Policy information about availability of computer code

Data collection	IncuCyte® ZOOM 2018A
Data analysis	Fiji 2.0.0-rc-69/1.52p<br>Graphpad Prism 6 for Mac

For manuscripts utilizing custom algorithms or software that are central to the research but not yet described in published literature, software must be made available to editors and reviewers. We strongly encourage code deposition in a community repository (e.g. GitHub). See the Nature Research guidelines for submitting code & software for further information.

Data

Policy information about availability of data

All manuscripts must include a data availability statement. This statement should provide the following information, where applicable:

- Accession codes, unique identifiers, or web links for publicly available datasets
- A list of figures that have associated raw data
- A description of any restrictions on data availability

No datasets were generated during the current study. CCLE dataset was analyzed: https://portals.broadinstitute.org/ccle/. Cbioportal was used for coexpression analyses: https://www.cbioportal.org/, dataset is Pancreatic Adenocarcinoma (TCGA, PanCancer Atlas)

Field-specific reporting

Please select the one below that is the best fit for your research. If you are not sure, read the appropriate sections before making your selection.

- [x] Life sciences
- [ ] Behavioural & social sciences
- [ ] Ecological, evolutionary & environmental sciences

For a reference copy of the document with all sections, see nature.com/documents/nr-reporting-summary-flat.pdf

Life sciences study design

All studies must disclose on these points even when the disclosure is negative.

Sample size	No sample size calculations were performed. Sample size was determined to be adequate based on the magnitude and consistency of measurable differences between groups, which was established from previous published works.
Data exclusions	On principle, data were only excluded for failed experiments, reasons for which included microbial contamination.
Replication	Experiments are routinely performed at least twice. All replicate experiments were successful.
Randomization	Pancreatic tumor-bearing mice were randomized into treatment arms for in vivo BMP1 inhibitor treatment studies. All groups in the in vitro treatment experiments were performed side-by-side under the same condition until treatment was added, samples were randomly assigned with groups.
Blinding	Investigators were blinded to treatment groups during the collection and analysis of all in vivo data. This included tumor weight measurements, metastasis load measurements as well as the immunohistological staining and quantifications.

Reporting for specific materials, systems and methods

We require information from authors about some types of materials, experimental systems and methods used in many studies. Here, indicate whether each material, system or method listed is relevant to your study. If you are not sure if a list item applies to your research, read the appropriate section before selecting a response.

Materials & experimental systems

n/a	Involved in the study	
[ ]	[x]	Antibodies
[ ]	[x]	Eukaryotic cell lines
[x]	[ ]	Palaeontology and archaeology
[ ]	[x]	Animals and other organisms
[x]	[ ]	Human research participants
[x]	[ ]	Clinical data
[x]	[ ]	Dual use research of concern

Methods

n/a	Involved in the study	
[x]	[ ]	ChIP-seq
[x]	[ ]	Flow cytometry
[x]	[ ]	MRI-based neuroimaging

Antibodies

Antibodies used

C-pro/a1(I) (600-401-D19, RL, Rockland); lot 37143
C-pro/a1(I) (LF42, Kerafast); homemade by Larry W. Fisher
VIM (ab92547, Abcam); lot GR219216-5
lamin A/C (ab108595, Abcam); lot GR257223-12
human CollI/a1(I) (AF6220, R&D systems);
human CollI/a2(I) (A5786, Abclonal); lot 1151680201
mouse CollI (AB765P, Millepore); lot 2328311
BMP1 (ab118520, Abcam); lot AB_10899275
Ki67 (SP6, VALENT); lot 051519
Cleaved caspase 3 (5A1E, Cell Signaling); lot 21
a2(I) (A16699, Abclonal); lot 1151680101
GAPDH (MAB374, Millipore); lot 2145925
N-pro/a1(I) (LF39, Kerafast); homemade by Larry W. Fisher
PCOLCE (A15298, Abclonal); lot 0112230201
b-actin (14-4); homemade by the Hynes lab

Validation

The antibodies used in these studies are well-characterized, validated commercial reagents from reliable sources (e.g., Abcam, Millepore) or generated in the lab and reported in the scientific literature: PMID: 29730502. Validation details for the commercially available antibodies are at the following links:
C-pro/a1(I) (600-401-D19, RL, Rockland): WB validation https://rockland-inc.com/Product.aspx?id=40819

	C-pro/a1(I) (LF42, Kerafast): see reference PMID: 8553864.<br>VIM (ab92547, Abcam): knockout validated https://www.abcam.com/vimentin-antibody-epr3776-cytoskeleton-marker-ab92547.html<br>lamin A/C (ab108595, Abcam): knockout validated, https://www.abcam.com/lamin-a--lamin-c-antibody-epr4100-nuclear-envelope-marker-ab108595.html<br>human CollI/a1(I) (AF6220, R&D systems): https://www.rndsystems.com/products/human-collagen-i-alpha1-antibody_af6220<br>human CollI/a2(I) (A5786, Abclonal): https://abclonal.com/catalog-antibodies/COL1A2PolyclonalAntibody/A5786<br>mouse CollI (AB765P, Millepore): validated for WB, IHC, IF. https://www.merckmillipore.com/CN/zh/product/Anti-Mouse-Collagen-Type-I-Antibody,MM_NF-AB765P?ReferrerURL=https%3A%2F%2Fcn.bing.com%2F&bd=1<br>BMP1 (ab118520, Abcam): https://www.abcam.com/bmp1pcp-antibody-ab118520.html<br>Ki67 (SP6, VALENT): https://biocare.net/product/ki-67-rabbit-antibody/<br>Cleaved caspase 3 (5A1E, Cell Signaling): https://www.cellsignal.com/products/primary-antibodies/cleaved-caspase-3-asp175-5a1e-rabbit-mab/9664<br>a2(I) (A16699, Abclonal): https://abclonal.com/catalog-antibodies/COL1A2PolyclonalAntibody/A16699<br>GAPDH (MAB374, Millipore): https://www.emdmillipore.com/US/en/product/Anti-Glyceraldehyde-3-Phosphate-Dehydrogenase-Antibody-clone-6C5,MM_NF-MAB374<br>N-pro/a1(I) (LF39, Kerafast): see reference PMID: 8553864.<br>PCOLCE (A15298, Abclonal): https://abclonal.com/catalog-antibodies/PCOLCEPolyclonalAntibody/A15298<br>b-actin (14-4): generated in the lab and reported in the scientific literature: PMID: 29730502

Eukaryotic cell lines

Policy information about cell lines

Cell line source(s)	The human pancreatic adenocarcinoma (PDAC) cell lines AsPC1, BxPC3, PANC1 and MIAPaCa2 and HEK 293FT cells were purchased from American Type Cell Culture (ATCC); PSN1 and CFPAC1 were gifts from the Koch Institute cell line repository. The human CAF cell line hT1 and hM1 were gifts from Dr. David Tuveson laboratory and published previously REF59.
Authentication	The human pancreatic adenocarcinoma (PDAC) cell lines AsPC1, BxPC3, PANC1 and MIAPaCa2 and HEK 293FT cells were purchased from American Type Cell Culture (ATCC), where they were tested and authenticated with STR profiling; PSN1 and CFPAC1 were gifts from the Koch Institute cell line repository, they were deposited after arrival from ATCC, where they were tested and authenticated with STR profiling.
Mycoplasma contamination	Once the cells are cultured in our laboratories, they were screened bimonthly for mycoplasma contamination using a commercially available mycoplasma PCR detection kit (ATCC, Cat# 30-1012K). All experiments were performed with mycoplasma negitive cells.
Commonly misidentified lines<br>(See ICLAC register)	No cell lines used are listed in the database of commonly misidentified cell lines.

Animals and other organisms

Policy information about studies involving animals; ARRIVE guidelines recommended for reporting animal research

Laboratory animals	8-10-week-old NOD/SCID/IL2Rγ-null (NSG) male mice were used for orthotopic and tail vein transplantation experiments.
Wild animals	This study did not involve wild animals.
Field-collected samples	This study did not involve samples collected from the field.
Ethics oversight	All experiments were conducted in accordance with procedures approved by the MIT Animal Care and Use Committees.

Note that full information on the approval of the study protocol must also be provided in the manuscript.
